# A Mixed Longitudinal EEG Study of Sensorimotor Rhythm Modulation and Its Relationship with Language Development in Children Aged 3–10 Years

**DOI:** 10.3390/brainsci16080880

**Published:** 2026-08-18

**Authors:** Vladimir Lipatov, Anna Rebreikina, Olga Sysoeva

**Affiliations:** 1 Centre for Research on Talent Development, Sirius University of Science and Technology, Sirius Federal Territory, 354340 Sochi, Russia; chubakapetrovich@gmail.com (V.L.); olga.v.sysoeva@gmail.com (O.S.); 2 Laboratory of Human Higher Nervous Activity, Institute of Higher Nervous Activity and Neurophysiology of RAS, 117485 Moscow, Russia

**Keywords:** EEG, sensorimotor rhythm, longitudinal study, language development, preschool and primary school children

## Abstract

**Background:** Sensorimotor (mu) rhythms reflect the functional state of sensorimotor cortical networks and are of increasing interest for understanding typical and atypical neurodevelopment. However, the developmental trajectories of mu rhythm modulation in preschool and early school-age children remain poorly characterized. **Objectives**: We studied age-related changes in alpha (8–13 Hz) and beta (13–30 Hz) sensorimotor rhythms in children aged 3 to 10 years using a mixed longitudinal design and investigated their relationship with language development. **Methods:** EEG was recorded twice (interval ~1 year) in 44 typically developing children during three conditions: passive hand movement (PHM), video hand movement observation (VHM), and a control condition (video fractal movement, VFM). Language was assessed with the Preschool Language Scales—Fifth Edition (PLS-5) in a subset of 32 of the 44 participants at the first time point. Modulation indices (log10(experimental/control)) were computed, and repeated-measures ANOVAs and Spearman correlations were performed. **Results**: PHM elicited desynchronization in both alpha and beta bands, while VHM induced alpha synchronization only. Alpha desynchronization showed contralateral lateralization during right- and left-hand movements, without age-related changes. Beta desynchronization showed no lateralization. Beta desynchronization during PHM correlated negatively with Auditory Comprehension and Total Language scores (ρ up to −0.62, FDR-corrected *p* < 0.05), indicating that more pronounced desynchronization of sensorimotor rhythms relates to better language abilities. **Conclusions**: These findings support the involvement of sensorimotor networks in auditory language comprehension and suggest that beta mu rhythm may serve as a sensitive marker of individual differences in language development, though replication in larger samples is warranted.

## 1. Introduction

Sensorimotor (mu) rhythms represent bioelectrical brain activity recorded predominantly over sensorimotor areas with the most pronounced modulation (suppression) occurring in conditions associated with motor activity [1]. Two main components of sensorimotor rhythms are distinguished according to their frequency range, topographic dominance, and functional properties. The alpha component of the sensorimotor rhythm is recorded in the 8–13 Hz range, manifests predominantly in the postcentral somatosensory cortex [2], and primarily reflects sensorimotor function. The beta component of the sensorimotor rhythm is recorded in the 13–30 Hz range, mainly in the precentral motor cortex, and is more closely associated with motor preparation and execution [3].

In children, sensorimotor rhythm is recorded as early as the first months of life; the alpha component of the mu rhythm is already observed in infancy [4]. The most pronounced increase in sensorimotor rhythm frequency occurs during the first year of life: at 11 weeks of age, movement-related mu rhythm modulation can be recorded at a frequency of ~2.75 Hz, whereas by 47 weeks the mu rhythm frequency rises to 8.25 Hz; thereafter, the increase slows down, and by 4 years of age mu rhythm modulation can be recorded in the 9 Hz range [5]. In adolescence, the modulation frequency stabilizes at approximately 10 Hz [6].

This rapid increase in sensorimotor rhythm frequency during the first year of life is associated with general brain development: active neuronal myelination, the formation and maturation of structural and functional characteristics of cortical networks, and changes in the density and orientation of neuronal ensembles [5]. In contrast to frequency characteristics, the amplitude characteristics of sensorimotor rhythms decrease with age, which is related to an increase in skull thickness and a corresponding rise in tissue resistance [7].

The majority of studies in children to date have focused on infants and children up to 3 years of age [8]. At the same time, age-related features of sensorimotor activity in preschool and early school-age children remain understudied, although this developmental period is characterized by high individual variability and functional immaturity of brain bioelectrical activity as well as sensorimotor function.

Moreover, the vast majority of mu rhythm studies in children have focused exclusively on the alpha component of the sensorimotor rhythm [5,7,9]. A few studies have examined the beta component of the sensorimotor rhythm in children. The magnetoencephalography (MEG) studies either covered two discrete age groups of children (4–6 and 11–13 years), thus excluding children of early school age (7–10 years) [10], or examined adolescents aged 9 to 15 years [11]. Electroencephalography (EEG) studies of the beta component in children analyzed only central channels (C3, C4, Cz) [12,13]. Thorpe and colleagues [14] questioned the very existence of the beta component of the sensorimotor rhythm in infants and young children. In their study, a significant level of event-related desynchronization in the beta range (15–25 Hz) was observed only in the adult group and was absent in 4-year-old children, leading the authors to suggest that the beta component of the sensorimotor rhythm develops after the age of 4. Overall, despite a large body of studies on sensorimotor activity, the issue of the developmental dynamics of sensorimotor rhythm desynchronization in both frequency bands remains a matter of debate. Studies have shown both an increase in desynchronization strength with age [9,15] and an absence of age-related changes [7].

The topography of sensorimotor rhythm suppression in children also requires further investigation. Although sensorimotor rhythms are traditionally analyzed at central sites overlaying sensorimotor cortex activity, several studies have also reported mu rhythm suppression in parietal regions [14]. In the beta range, suppression has also been observed in frontal areas in various tasks [16]. Of particular interest are age-related changes in interhemispheric asymmetry of sensorimotor rhythms. In adults, suppression of the alpha mu rhythm is typically observed in the hemisphere contralateral to the moving hand (for a review, see [1]); in children, however, the lateralization has been less thoroughly studied and has primarily been investigated during right-hand movement. In children aged 4 to 14 years, right-hand manipulation has also elicited pronounced mu rhythm suppression in the contralateral (left) hemisphere, while suppression in the ipsilateral (right) hemisphere has not been significant [7]. However, the developmental dynamics of this asymmetry have not been examined. Given that handedness continues to consolidate throughout the preschool period [17], we hypothesize that this process may be accompanied by changes in the functional asymmetry of somatosensory activity.

Many researchers consider sensorimotor rhythms as potential neurophysiological markers of development [18,19], indicators of motor neural network formation [20]. In this regard, studies suggesting the involvement of sensorimotor networks in speech and language processes are of great interest. There is growing evidence that speech perception engages an extensive multisensory network that includes sensorimotor areas of the brain [21,22]. According to a study by Saltukalaroglu and colleagues, sensorimotor rhythms can serve as indicators of time-varying sensorimotor integration processes during speech perception and production [23]. In adulthood, studies show sensorimotor system involvement in the perception of action-related words, as well as in speech perception under noisy conditions, that is, conditions in which speech is more difficult to discern [23,24]. In children, a link between sensorimotor rhythms and speech perception has also been identified: for example, Patzwald and colleagues found that more pronounced mu rhythm desynchronization was observed in children with a larger receptive repertoire of active verbs [25], as well Mikhailova and colleagues found that children with a higher level of language comprehension exhibited more pronounced mu rhythm desynchronization compared to children with an average level of language comprehension [26]. However, the limited pool of existing studies examining the relationship between sensorimotor rhythms and language in children has largely focused on infancy and early childhood (the first three years of life), predominantly considering only the alpha component of the mu rhythm [26,27].

Thus, the age range of preschoolers and early school-age children (i.e., 3–10 years) remains almost unexamined in terms of the sensorimotor rhythm characteristics and developmental dynamics, even though active development of motor skills, cognitive functions, and language continues during this age period. Despite the general patterns in the development of these functions, individual trajectories of their development can vary quite significantly. The bioelectrical activity is also characterized by high interindividual variability in this age range. Even within a single year, the brain’s bioelectrical activity, as well as psychomotor and cognitive functions and speech, can undergo significant changes. Given the ongoing maturation of neural systems and the substantial heterogeneity in developmental trajectories, a mixed longitudinal design appears to be the most optimal approach for studying age-related effects. The cross-sectional component of the analysis allows for capturing general age-related changes across the period from 3 to 10 years, while the one-year longitudinal analysis allows for a more precise reflection of each participant’s individual developmental dynamics within a relatively short study period. This mixed longitudinal approach allows us to capture age-related differences across the 3–10 year span within a single year of testing by including multiple age cohorts, while the one-year follow-up provides insight into individual developmental trajectories. We assume that such a combination of approaches may help identify the age period during which the most pronounced developmental changes in sensorimotor activity occur within the studied age range.

The aim of the present research is to study age-related characteristics of alpha and beta component modulation of sensorimotor rhythms in children aged 3 to 10 years during the inclusive passive hand movement paradigm (PHM) and the video hand movement paradigm (VHM) within a mixed longitudinal design. We selected these experimental paradigms based on their potential for future use in children with developmental disorders who have difficulty performing voluntary movements or following instructions. An additional objective of the present study is to examine the relationship between sensorimotor rhythm modulation and language development. While the cited studies often focus on the perception of spoken speech, in the present work we focus on general language abilities, as measured by standardized language scales, to capture the broader relationship between sensorimotor network function and linguistic competence.

In accordance with the stated aims of the study, the following research tasks were formulated. First, we aimed to study the characteristics of sensorimotor rhythms in children aged 3 to 10 years and examine the topography of modulation of the alpha and beta components of sensorimotor rhythms and their lateralization. Second, we aimed to examine the age-related dynamics of sensorimotor rhythm modulation over a one-year period and across different age groups. Third, we aimed to study the relationship between sensorimotor rhythms and language development in children.

Thus, the corresponding hypotheses are: (1) Sensorimotor rhythm modulation increases with age during the preschool and early school-age years. (2) An increase in the lateralization of sensorimotor activity during right- or left-hand movements will be observed with age. (3) Sensorimotor rhythm is associated with language development in preschool and early school-age children.

## 2. Materials and Methods

### 2.1. Participants

The present study is part of a larger longitudinal research project “Mechanisms of sensory integration in oral and written language development” of the Sirius University of Science and Technology. For the present study, 56 children aged 3 to 10 years who had already completed both stages of the longitudinal study within the research project were selected. Twelve children were excluded from the study: two were excluded due to incomplete participation in the experimental paradigms, and ten were excluded due to excessive noise and artifacts in the EEG recordings. The final sample comprised 44 children (24 boys, 20 girls; age at the first longitudinal time point: 3.58–8.83 years, M = 6.12 ± 1.29; age at the second longitudinal time point: 4.75–10.16 years, M = 7.34 ± 1.30). A post hoc sensitivity power analysis, reported in the Limitations section, was conducted to determine the minimum effect sizes detectable given the achieved sample size. The mean interval between the first and second longitudinal time points was one year and two months (M = 14.65 ± 2.92 months).

To examine age-related characteristics of sensorimotor rhythms, the final sample was divided into three groups based on age at the first longitudinal time point: children aged 3 years to 4 years 11 months (*n* = 8); children aged 5 years to 6 years 11 months (*n* = 23); and children aged 7 years to 8 years 11 months (*n* = 13). This subgroup division ensured a comparable two-year time interval across groups.

Additionally, a parental questionnaire was included in the study to assess hand dominance in children. Hand dominance was assessed with 18 questions about preferred hand in various everyday situations; each item was scored using a scale from −2 (always uses the left hand) to 2 (always uses the right hand). The total score was calculated as the average across all items. Participants who scored more than 0.7 points were considered right-handed, participants who scored between −0.7 and 0.7 points were considered ambidextrous, and participants who scored less than −0.7 points were considered left-handed. According to the questionnaire results, the sample comprised 34 right-handed children, 2 left-handed children, 5 ambidextrous children, and 3 children whose parents did not complete the questionnaire.

This study was approved by the Sirius University Bioethics Committee (Bioethics Committee Opinion of 13 July 2022) in accordance with the Declaration of Helsinki. Participants’ parents signed informed consent, and children also gave verbal assent for the study. This study followed the approved protocols of EEG and behavioral data collection and management.

### 2.2. Language Assessment

Children’s language development was assessed at the first longitudinal time point using the Russian version of the standardized psychometric instrument, the Preschool Language Scales—Fifth Edition (PLS-5). This instrument was selected because it is an international, standardized tool that has been translated into Russian [28]. Only 32 participants from the final study sample completed the PLS-5; data from 12 participants were excluded because the PLS-5 was administered with a significant delay relative to the EEG recording. The following age-standardized scores were used in the analysis: the Total Language score, which reflects overall language ability, and the Auditory Comprehension and Expressive Communication standard scores.

### 2.3. Stimulus Material

Passive Hand Movement (PHM)

The experimental condition of passive hand movement was adapted from a previous study [15] and consisted of an experimenter passively clenching and unclenching the child’s right and left hands 15 trials per hand in an alternating manner. Prior to the manipulation, the child was informed that their hand would be clenching and unclenching and was asked to look at their hand.

Video Hand Movement (VHM)

The stimulus material for the experimental condition of hand movement observation on video was a video clip showing a close-up view of hands bimanually manipulating brightly colored soap (manipulation of an object with salient visual characteristics was necessary to maintain attention in young children). The video demonstrated the following manipulations: holding the object and crushing the object (soap) with the fingers, shown in close-up.

Video Fractal Movement (VFM)

The video with fractals served as the control condition. According to K. Cuevas and colleagues, who provided practical recommendations for the study of sensorimotor rhythms in children, the presentation of meaningless, abstract visual stimuli can be used as a control condition, as such stimuli are not expected to induce mu rhythm modulation [29]. The video selected for the control condition contained abstract fractals on a black background, which moved slowly and changed several times throughout the video. Screenshots of the video stimuli used for the VFM and VHM paradigms are publicly available; the corresponding link is provided at the end of Section 2.5. EEG Acquisition and Preprocessing.

### 2.4. Experimental Procedure

**Figure 1:**
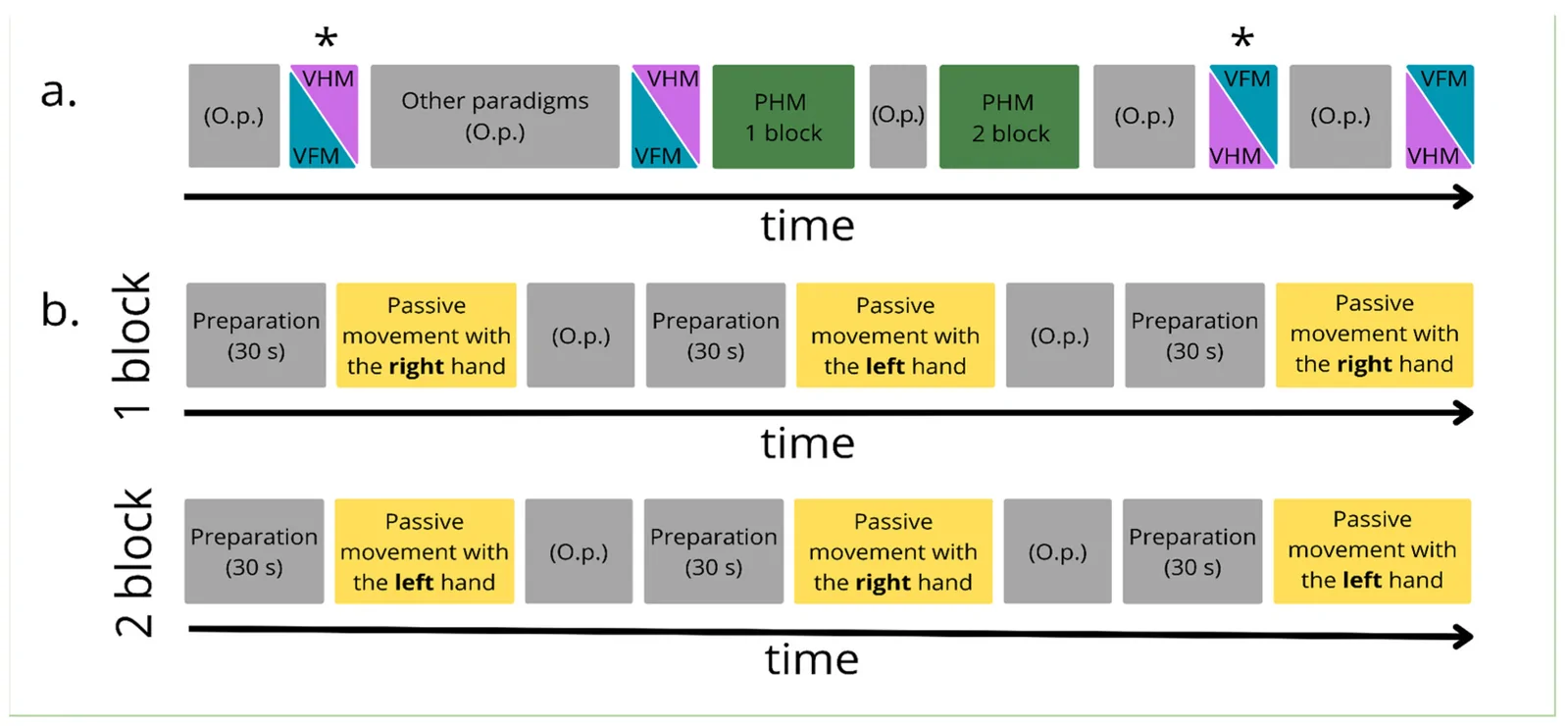
(**a**) General scheme of the experiment. The paradigms analyzed in this study are highlighted in color. Other paradigms (O.p.): paradigms not included in this study (marked in gray). The blue block (VFM): control condition “Video fractal movement”; the purple block (VHM): paradigm “Video hand movement”. (**b**) The scheme of Blocks 1 and 2 demonstrates the experimental procedure for the “Passive hand movement” paradigm (PHM). First, a preparation phase (instruction familiarization and brief tactile contact) was conducted, followed by the PHM condition for the right or left hand. * indicates paradigms that were not presented to participants (*n* = 12) at the first longitudinal time point, for whom the duration of the VHM and VFM paradigms was 30 s.

The neurophysiological stage of the study was conducted in two sessions, one year apart. The experimental procedure for the VHM and VFM conditions specifically was slightly modified over the course of data collection at the first longitudinal time point; the paradigm remained unchanged during the second longitudinal time point. Initially, each of the VHM and VFM conditions lasted 30 s (*n* = 12 participants completed this version of the paradigm; Mean age (months) = 66.00 ± 13.82; range 43.0–88.0). Subsequently, the presentation time was doubled by presenting the stimulus material twice, alternating with other experimental tasks that are not evaluated in the present study, resulting in a total duration of 60 s for each condition (*n* = 15; M = 67.40 ± 13.64; range 48.0–94.0). Finally, the 30-s segment itself was extended to 45 s, yielding a total duration of 90 s for each condition (*n* = 17; M = 84.12 ± 12.68; range 62.0–106.0). At the second longitudinal time point, the VHM and VFM paradigms remained unchanged, and each condition lasted 90 s for all participants. Prior to the PHM paradigm, an instruction slide was shown for 30 s (also read aloud) to familiarize the child with the task; during the instruction presentation, a brief tactile contact with the assessor’s hands was made to help the child habituate. The PHM paradigm was then performed, during which the assessor clenched and unclenched the child’s hand 15 times, while a black screen with an instruction was displayed on the monitor; the assessor performing the paradigm stood on the side of the hand being clenched. This was followed by a 45-seconds eyes-closed condition (this condition is not analyzed in the present study). A detailed scheme of the experiment is shown in Figure 1.

### 2.5. EEG Acquisition and Preprocessing

EEG recording was performed in a noise- and electrically isolated room using a 32-channel active electrode system (actiCHamp, Brain Products, Gilching, Germany) with a sampling rate of 50 kHz. The electrodes were placed according to the international extended 10–20 system. Electrode FCz was set as the reference. Impedances of most electrodes were maintained below ~25 kOhm. The EEG data were online band-pass filtered at 0.1–100 Hz.

The stimulus material was presented to the participant via a 23.8-inch LED monitor with a resolution of 1920 × 1080 pixels, positioned at a viewing distance of 60 cm from the participant. The experiment was conducted using Presentation software (v. 23.1.09.29.22), which synchronizes stimuli presentation with EEG and sends markers indicating the start and end of each paradigm to the EEG recording.

EEG analysis and preprocessing were performed using Python (version 3.8) in Jupyter Notebook (version 7.2.2), with the MNE-python package version 1.7.0 (https://mne.tools/ (accessed on 13 August 2026)). For raw data preprocessing, the sampling frequency of the EEG recording was reduced from 50 kHz to 500 Hz (the high initial sampling rate was required for other experimental conditions of the scientific project that are not examined in this paper). The EEG data were filtered with a 1–50 Hz band-pass filter. Channels with a large number of artifacts and high noise levels (identified manually) were interpolated. Segments of the EEG recording containing numerous artifacts were manually marked as bad epochs to prevent them from distorting the ICA results during subsequent analysis; these segments were also automatically rejected at later preprocessing stages. After interpolation, the data were cleaned of remaining artifacts using Independent Component Analysis (ICA) with the FastICA algorithm. Automatic labeling of independent components was performed using the machine-learning-based ALICE approach [30]. After that, for components automatically classified as ocular or muscle artifacts, subsequent manual confirmation was performed based on component topography and characteristic activity patterns. Next, EEG was re-referenced to average reference and segmented into 2-second epochs. Epochs containing unremoved high-amplitude artifacts and those previously marked as bad were automatically excluded using an amplitude threshold set at 200 μV. The proportion of rejected epochs does not vary considerably across experimental conditions. For the PHM condition, which comprised, on average, 45 epochs (every 2 s in duration), 7.5% of epochs were excluded. In the VHM paradigm, which consisted of 45 epochs, the proportion of rejected epochs was 7.3%. In the VFM condition, also consisting of 45 epochs, 10% of epochs were excluded.

Current source density (CSD) analysis was applied to the data using the recommended MNE settings (sphere = ‘auto’, lambda2 = 1 × 10^−5^, stiffness = 4, n_legendre_terms = 50). The purpose of applying the CSD transformation was to improve the spatial accuracy of the EEG data and to amplify local neural signal sources [31]. After that, a Fast Fourier Transform (FFT) was applied to the EEG recordings, which was used to calculate the mean signal amplitude for the 8–13 Hz and 13–30 Hz ranges, for all channels and experimental conditions. We used the 8–13 Hz frequency range for the alpha component of the sensorimotor rhythm across our studied age range based on a longitudinal study by Marshall study, which showed that alpha frequencies at central sites increased from 7–8 Hz at 10 and 24 months to 9 Hz at 4 years [32]; the Berchicci study also showed that sensorimotor rhythms in children approach these frequency values from approximately 3–4 years of age [5]. The script used for FFT calculation is available via the link provided at the end of this section. The final analysis included the following channels: F3, F4, FC1, FC2, FC5, FC6, C3, C4, CP1, CP2, CP5, CP6. This relatively wide set of channels (compared to the traditional C3 and C4) was justified by studies indicating that it is necessary to analyze not only bioelectrical activity at the central channel but also in fronto-parietal regions, where sensorimotor rhythm activity can also be observed [14].

The scripts and data used for this study can be found at the following link: https://osf.io/mnuaz/overview (accessed on 13 August 2026).

### 2.6. Statistical Analysis

Statistical analysis of the data was performed separately for 8–13 Hz and 13–30 Hz ranges using repeated measures analysis of variance (RM ANOVA) in the graphical interface for the R programming language—JASP (version 0.95.0.0). The following within-group factors were selected for the ANOVA: Longitudinal Time Point (levels: First TP, Second TP), Condition (levels: VFM, PHM, VHM), Hemisphere (levels: Left, Right) and Region (levels: F3/F4, FC5/FC6, FC1/FC2, C3/C4, CP5/CP6, CP1/CP2). The between-group factor was Age Group (levels: (3–4 years), (5–6 years), (7–8 years)), with age defined at the first longitudinal time point.

To investigate the lateralization of mu rhythm suppression during right- and left-hand clenching in the PHM condition, a RM ANOVA was performed for the second longitudinal time point only (due to technical issues with the labeling of right- and left-hand clenching in the first session, this measure could not be examined for that time point). The within-group factors were: Lateralization (VFM (control), PHM right hand, PHM left hand), Hemisphere (Left, Right) and Region (levels: F3/F4, FC5/FC6, FC1/FC2, C3/C4, CP5/CP6, CP1/CP2). The between-group factor was Age Group (levels: (3–4 years), (5–6 years), (7–8 years)), with age defined at the first longitudinal time point.

To examine the relationship between mu rhythm modulation and language development, the modulation index was calculated using the formula Log10(experimental condition/control condition (VFM)), using EEG data from the first longitudinal time point only, corresponding to the time point at which speech development was assessed. Correlation matrices were then computed for the 8–13 Hz and 13–30 Hz ranges between the modulation indices (PHM and VHM conditions) and the PLS-5 scores (Auditory Comprehension, Expressive Communication, Total Language).

## 3. Results

### 3.1. Sensorimotor Rhythms Characteristics

#### 3.1.1. 8–13 Hz Range

**Figure 2:**
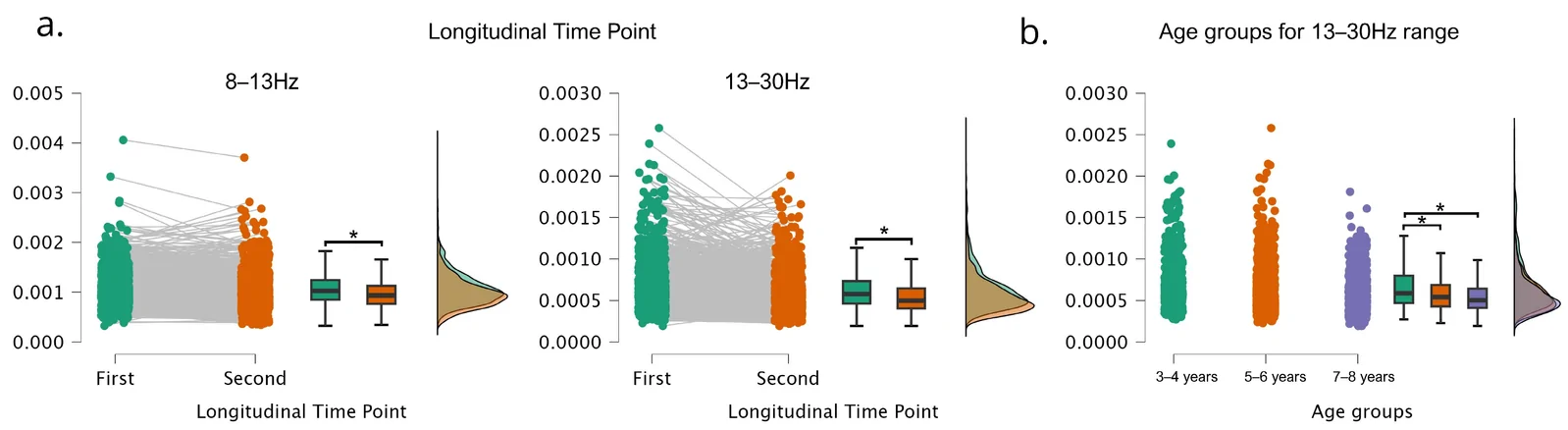
(**a**) Difference between the first and second longitudinal time points for the 8–13 Hz and 13–30 Hz ranges. Post hoc showed a significant difference between the first and second longitudinal time points, with amplitude being higher at the first time point across both frequency ranges, regardless of condition or channel. (**b**) Difference for the between-group factor Age Group (3–4 years-old age group; 5–6 years-old age group; 7–8 years-old age group at the first longitudinal time point) for the 13–30 Hz range. Post hoc for the factor Age Group for the 13–30 Hz range revealed significant differences between the 3–4 years-old age group and 5–6 years-old age group, as well as between the 3–4 years-old age group and 7–8 years-old age group. The y-axis represents mean amplitude (V/m^2^). Significant differences are marked with brackets and “*”.

The main effects were found for Longitudinal Time Point (F = 27.853, *p* < 0.001, *η*^2^*p* = 0.405), with overall amplitude values being higher at the First Longitudinal TP than at the Second (see Figure 2a). The complete RM ANOVA results for the 8–13 Hz range are presented in Supplementary Materials: Table S1.

**Figure 3:**
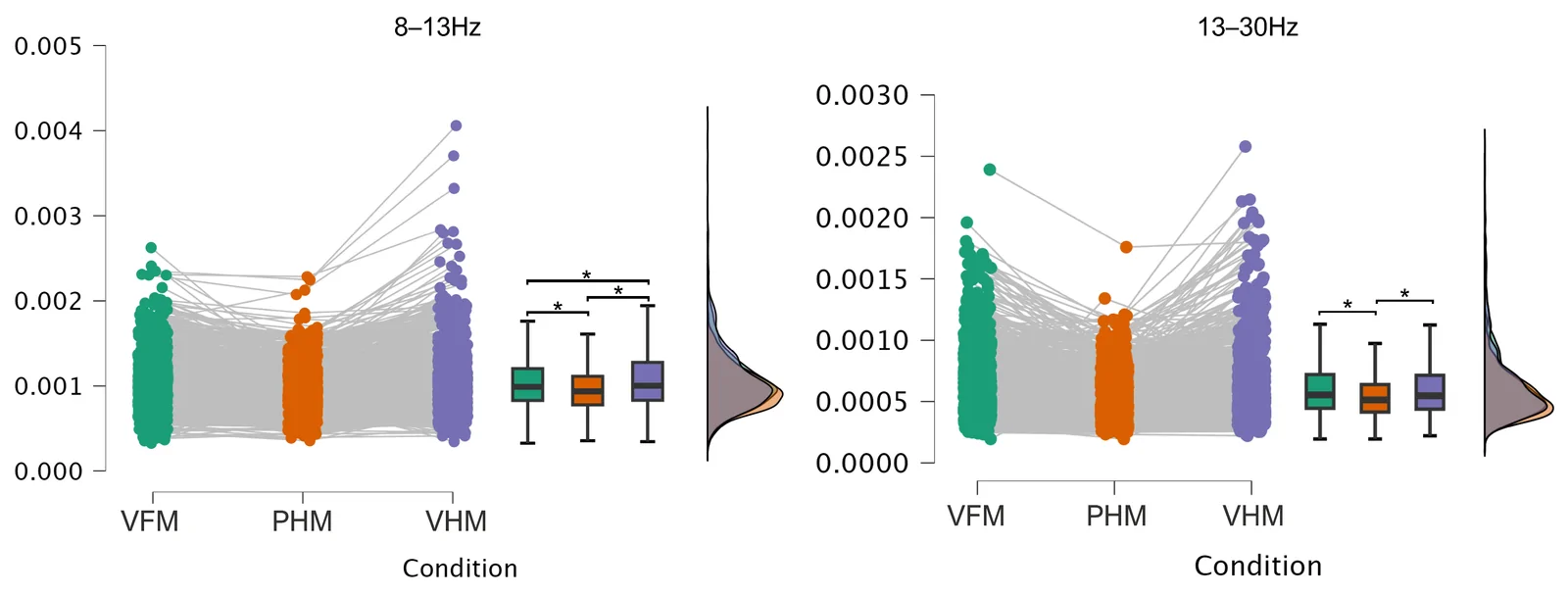
Differences between conditions (VFM, VHM, PHM) for the 8–13 Hz and 13–30 Hz ranges. For the 8–13 Hz range, significant differences were observed between the VFM condition and both experimental conditions (PHM and VHM), as well as between the two experimental conditions. For the 13–30 Hz range, significant differences were observed between the VFM condition and PHM, and between the two experimental conditions. The y-axis represents mean amplitude (V/m^2^). Significant differences are marked with brackets and “*”.

Factor Condition was also significant (F = 24.488, *p* < 0.001, *η*^2^*p* = 0.374), as well as its interaction with Region (F = 9.785, *p* < 0.001, *η*^2^*p* = 0.193). Post Hoc for the Condition factor identified significant differences between VFM and PHM (Cohen’s d = 0.285, t = 5.840, *p*(Holm) < 0.001), and VFM vs. VHM (Cohen’s d = −0.123, t = −2.148, *p*(Holm) = 0.038). In the PHM condition, the mu rhythm’s amplitude decreased compared to the VFM condition, indicating desynchronization. Conversely, in the VHM condition, the mu rhythm amplitude increased relative to the VFM, showing synchronization. Additionally, there were significant differences between PHM and VHM (Cohen’s d = −0.407, t = −5.721, *p*(Holm) < 0.001) (see Figure 3).

Post hoc for the Condition × Region interaction revealed that significant differences between VFM vs. PHM and PHM vs. VHM hold for frontocentral, central and central-parietal regions (FC1/FC2, C3/C4, CP1/CP2), while differences between VFT and VHM were not significant in these analyses, when regions were considered separately (see Figure S1 in Supplementary Materials and full Post Hoc for the Condition × Region in Table S1.3 in Supplementary Materials).

#### 3.1.2. 13–30 Hz Range

The main effects were found for Longitudinal Time Point (F = 50.144, *p* < 0.001, *η*^2^*p* = 0.550), with overall amplitude values being higher at the First Longitudinal TP than at the Second (see Figure 2a). A significant effect of the Hemisphere was found (F = 4.182, *p* = 0.047, *η*^2^*p* = 0.093), with higher amplitude in the right hemisphere than in the left hemisphere. The complete RM ANOVA results for the 13–30 Hz range are presented in Supplementary Materials: Table S2.

Significant Age Group effect (F = 4.746, *p* < 0.014, *η*^2^*p* = 0.188) was attributed to the difference between the 3–4 and 5–6 year-old groups (Cohen’s d = 0.531, t = 2.429, *p*(Holm) = 0.039), as well as between the 3–4 and 7–8 year-old groups (Cohen’s d = 0.727, t = 3.040, *p*(Holm) = 0.012). No significant difference was found between the 5–6 and 7–8 year-old groups (*p* = 0.294) (see Figure 2b).

**Figure 4:**
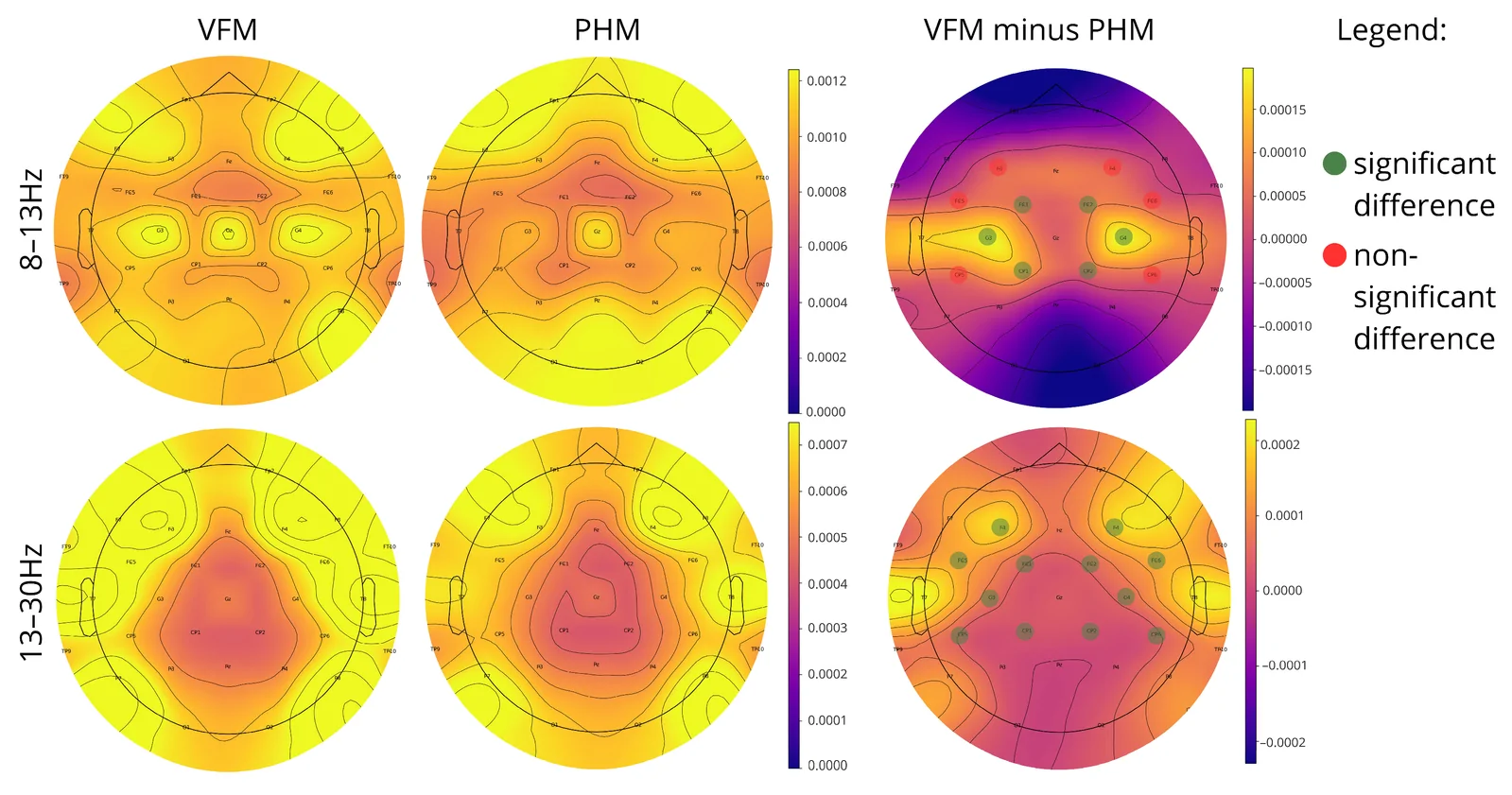
Topography of bioelectrical activity for the VFM and PHM conditions in the two frequency ranges. Demonstration of channels where significant differences between the VFM condition and PHM were observed.

Significant effects of Condition (F = 35.818, *p* < 0.001, *η*^2^*p* = 0.466) and its interaction with Region (F = 12.718, *p* < 0.001, *η*^2^*p* = 0.237) were also observed. Post-hoc analysis of the Condition effect revealed that passive hand movement resulted in a significant decrease in amplitude compared to the VFM condition (VFM vs. PHM: Cohen’s d = 0.491, t = 8.767, *p*(Holm) < 0.001) and VHM condition (VHM vs. PHM Cohen’s d = −0.468, t = −6.781, *p*(Holm) < 0.001). No significant difference was observed between the VFM condition and VHM (*p* = 0.746) (see Figure 3). Post hoc for the Condition × Region interaction revealed significant differences between the VFM and PHM across all analyzed regions (see Figure 4), and significant differences between the PHM and VHM experimental conditions at F3/F4, FC5/FC6, FC1/FC2, C3/C4, CP5/CP6 (see full Post Hoc for the Condition × Region in Table S2.3 in Supplementary Materials).

There was also significant Age Group × Condition × Region interaction (F = 1.865, *p* = 0.014, *η*^2^*p* = 0.083). To follow up on this interaction, post hoc RM ANOVAs were conducted separately for each age group of children (with factors: Condition, Region) within this frequency range.

For the 3–4 age group, main effects were found for Condition (F = 14.77, *p* < 0.001, *η*^2^*p* = 0.678), and a significant interaction Condition × Region was also identified (F = 6.114, *p* < 0.001, *η*^2^*p* = 0.466). Post Hoc for the Condition factor identified significant differences between VFM vs. PHM (Cohen’s d = 0.812, t = 4.880, *p*(Holm) = 0.005), and PHM vs. VHM (Cohen’s d = −0.729, t = −4.159, *p*(Holm) = 0.008). Post hoc for the Condition × Region interaction showed significant differences between the VFM vs. PHM in the FC1/2 channels (Cohen’s d = 0.252, t = 7.623, *p*(Holm) = 0.017).

For the 5–6 age group, main effects were found for Condition (F = 13.99, *p* < 0.001, *η*^2^*p* = 0.389), and a significant interaction Condition × Region was also identified (F = 5.945, *p* < 0.001, *η*^2^*p* = 0.213). Post Hoc for the Condition factor identified significant differences between VFM vs. PHM (Cohen’s d = 0.445, t = 5.625, *p*(Holm) < 0.001), and PHM vs. VHM (Cohen’s d = −0.542, t = −4.381, *p*(Holm) < 0.001). Post hoc for the Condition × Region interaction showed significant differences between the VFM vs. PHM conditions at the F3/4 (Cohen’s d = 1.190, t = 4.384, *p*(Holm) = 0.012) and at the C3/4 (Cohen’s d = 0.299, t = 4.465, *p*(Holm) = 0.010) channels, and between the PHM vs. VHM conditions at the FC5/6 (Cohen’s d = −0.848, t = −3.755, *p*(Holm) = 0.045), C3/4 (Cohen’s d = −0.421, t = −3.797, *p*(Holm) = 0.042) and CP5/6 (Cohen’s d = −0.285, t = −3.725, *p*(Holm) = 0.047) channels.

For the 7–8 age group, main effects were found for Condition (F = 6.929, *p* < 0.004, *η*^2^*p* = 0.366). Post Hoc for the Condition factor identified significant differences between VFM vs. PHM (Cohen’s d = 0.544, t = 3.691, *p*(Holm) = 0.009), and PHM vs. VHM (Cohen’s d = −0.454, t = −3.278, *p*(Holm) = 0.013). The Condition × Region interaction was not significant (*p* = 0.157), pointing to uniform differences between conditions regardless of region.

### 3.2. Lateralization Effects During Right- and Left-Hand Clenching

#### 3.2.1. 8–13 Hz Range

The main effects in the 8–13 Hz range were found for Lateralization (F = 15.543, *p* < 0.001, *η*^2^*p* = 0.275) and Age Group (F = 3.831, *p* = 0.030, *η*^2^*p* = 0.157), as well as significant interactions: Lateralization × Hemisphere (F = 14.408, *p* < 0.001, *η*^2^*p* = 0.260) and Lateralization × Hemisphere × Region (F = 5.557, *p* < 0.001, *η*^2^*p* = 0.119). The complete RM ANOVA results for lateralization in the 8–13 Hz range are presented in Supplementary Materials: Table S3.1.

**Figure 5:**
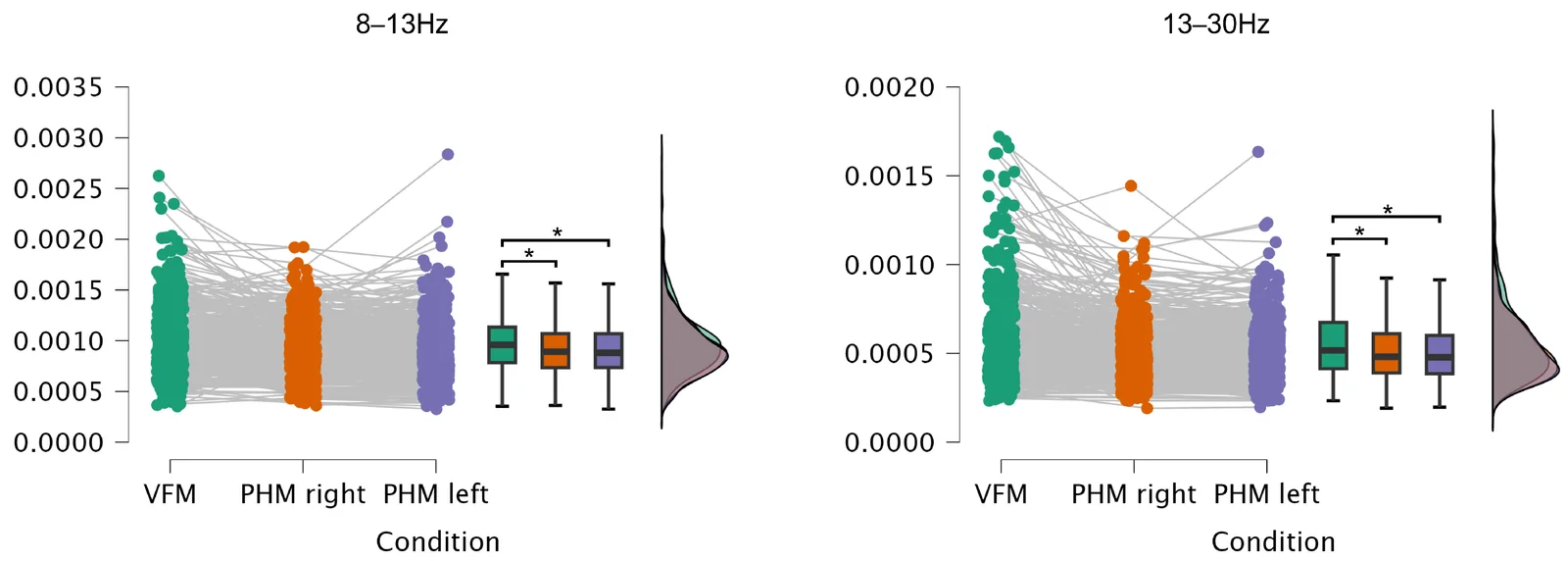
Differences between conditions (VFM, PHM right, PHM left) for the 8–13 Hz and 13–30 Hz ranges. In both frequency ranges, significant differences were observed between the VFM condition and the passive right-hand and left-hand movement conditions. The y-axis represents mean amplitude (V/m^2^). Significant differences are marked with brackets and “*”.

Significant effect of lateralization revealed a decrease in 8–13 Hz amplitude for both right-hand (Cohen’s d = 0.327, t = 4.229, *p*(Holm) < 0.001) and left-hand clenching (Cohen’s d = 0.306, t = 4.013, *p*(Holm) < 0.001) as compared to VFM (see Figure 5).

**Figure 6:**
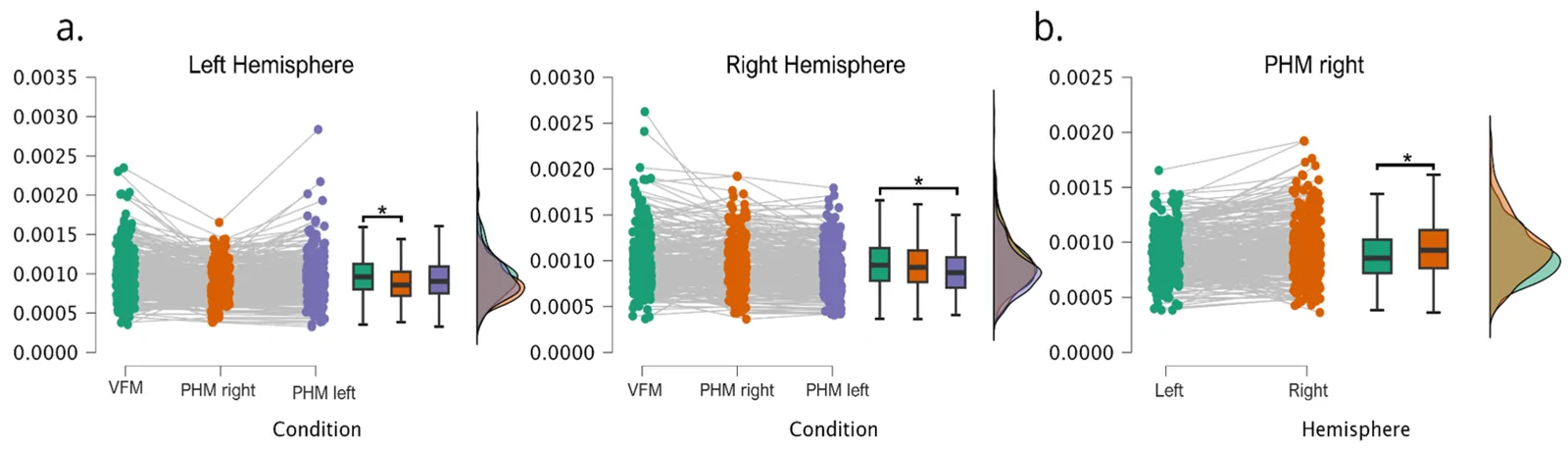
(**a**) Interaction between conditions (VFM, PHM right, and PHM left) in the right Figure 6. (**a**) Interaction between conditions (VFM, PHM right, and PHM left) in the right and left hemispheres for the 8–13 Hz range. (**b**) Difference between the right and left hemispheres for the PHM right condition for the 8–13 Hz range. The y-axis represents mean amplitude (V/m^2^). Significant differences are marked with brackets and “*”.

Following significant Lateralization × Hemisphere interactions, we revealed that during right-hand clenching (PHM right), the amplitude in the left hemisphere was significantly lower than the amplitude during the VFM condition both in the left hemisphere (Cohen’s d = −0.503, t = −4.643, *p*(Holm) < 0.001) and in the right hemisphere (see Figure 6a). On the contrary, amplitude in the right hemisphere in PHM right did not differ significantly from amplitude in either the right or left hemisphere in the VFM condition. Significant interhemispheric differences were also observed (Cohen’s d = −0.328, t = −4.083, *p*(Holm) = 0.002), with mu rhythm amplitude being greater in the right hemisphere than in the left in PHM right (see Figure 6b).

During left-hand clenching (PHM left), amplitude in the right hemisphere significantly differed from amplitude in the VFM condition in both the right (Cohen’s d = 0.409, t = 4.947, *p*(Holm) < 0.001) and the left hemispheres (Cohen’s d = 0.384, t = 4.676, *p*(Holm) < 0.001) (see Figure 6a). At the same time, no significant differences were observed between amplitude in the left hemisphere in PHM left and amplitude in the VFM condition in either the left or right hemisphere, and no statistically significant interhemispheric differences in PHM left were found.

**Figure 7:**
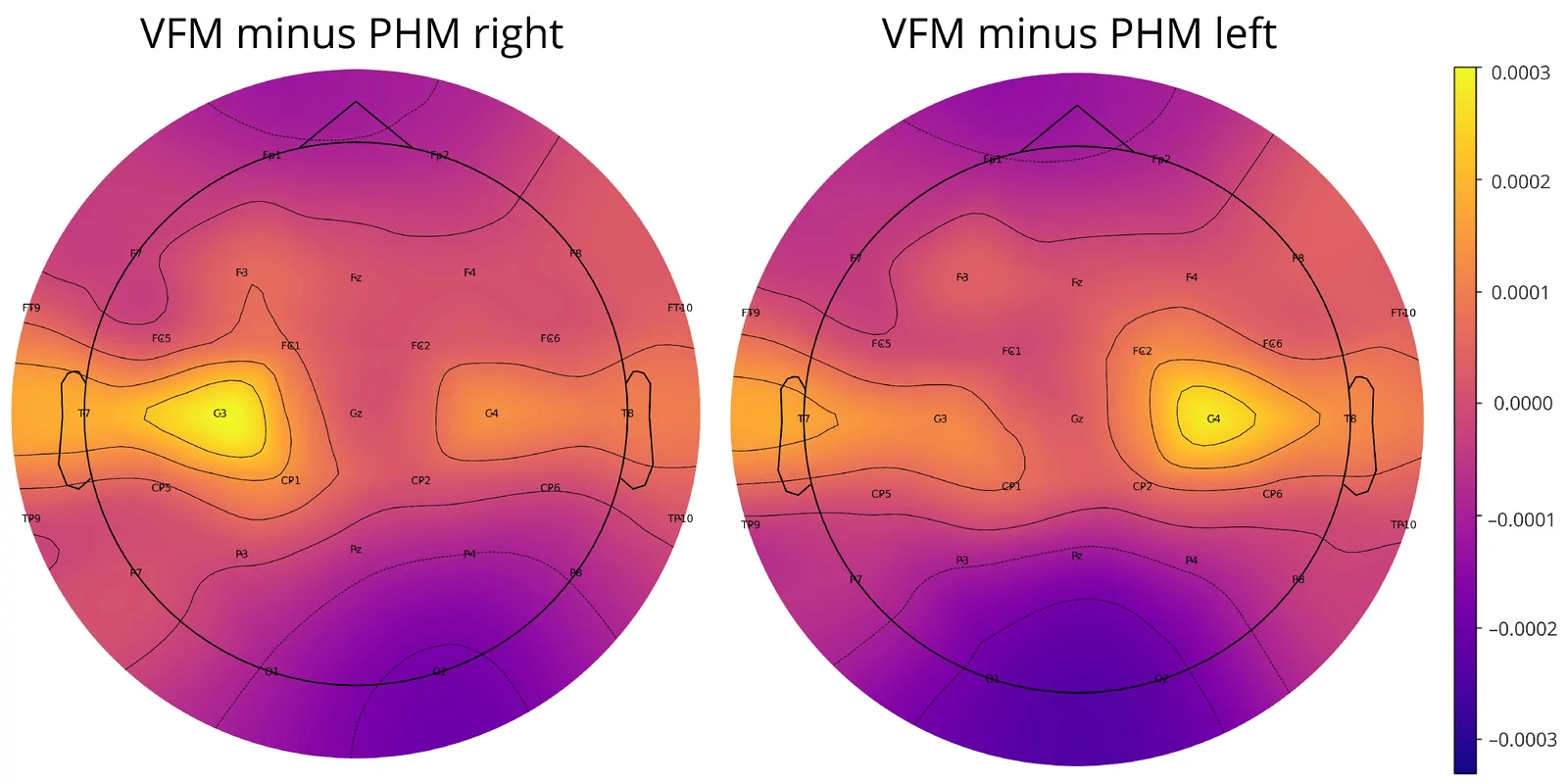
Topography of brain bioelectrical activity for the difference between the VFM condition and PHM for the right and left hands for the 8–13 Hz range.

Significant interactions Lateralization × Hemisphere × Region revealed that contralateral desynchronization during passive hand movement were significant in the central electrodes (in channel C3 between conditions VFM vs. PHM right (Cohen’s d = 1.290, t = 5.783, *p*(Holm) < 0.001), and in channel C4 between conditions VFM vs. PHM left (Cohen’s d = 1.053, t = 5.398, *p*(Holm) = 0.002)), with desynchronization during passive left hand movement being significant also in channel FC2 (VFM vs. PHM left (Cohen’s d = 0.533, t = 4.554, *p*(Holm) = 0.020) and PHM right vs. PHM left (Cohen’s d = 0.368, t = 4.971, *p*(Holm) = 0.006),) (see full Post Hoc for the Lateralization × Hemisphere × Region in Table S3.1.4 in Supplementary Materials). The topography of differences in bioelectrical activity between the VFM condition and passive clenching of the right and left hand in the 8–13 Hz range is presented in Figure 7.

Significant effects of Age Group revealed differences between the 3–4 years-old age group and 7–8 year-old age group (Cohen’s d = 0.931, t = 2.768, *p*(Holm) = 0.025). Specifically, children in the younger group exhibited higher amplitude and, consequently, higher amplitude compared to children in the older group, regardless of other factors.

#### 3.2.2. 13–30 Hz Range

Significant main effects in the 13–30 Hz range were found for Lateralization (F = 38.646, *p* < 0.001, *η*^2^*p* = 0.485) and Age Group (F = 4.239, *p* = 0.021, *η*^2^*p* = 0.171). The complete RM ANOVA results for lateralization in the 13–30 Hz range are presented in Supplementary Materials: Table S3.2.

A significant effect of lateralization revealed a similar decrease in 13–30 Hz amplitude for both right-hand (Cohen’s d = 0.473, t = 6.327, *p*(Holm) < 0.001) and left-hand clenching (Cohen’s d = 0.530, t = 6.797, *p*(Holm) < 0.001) as compared to the VFM condition (see Figure 5). Also, similarly to the results obtained for the 8–13 Hz range, a significant difference in amplitude was found between the 3–4 years-old age group and the 7–8 year-old age group (Cohen’s d = 0.788, t = 2.888, *p*(Holm) = 0.018).

### 3.3. Relationship Between Sensorimotor Rhythm Modulation Index and Language

**Figure 8:**
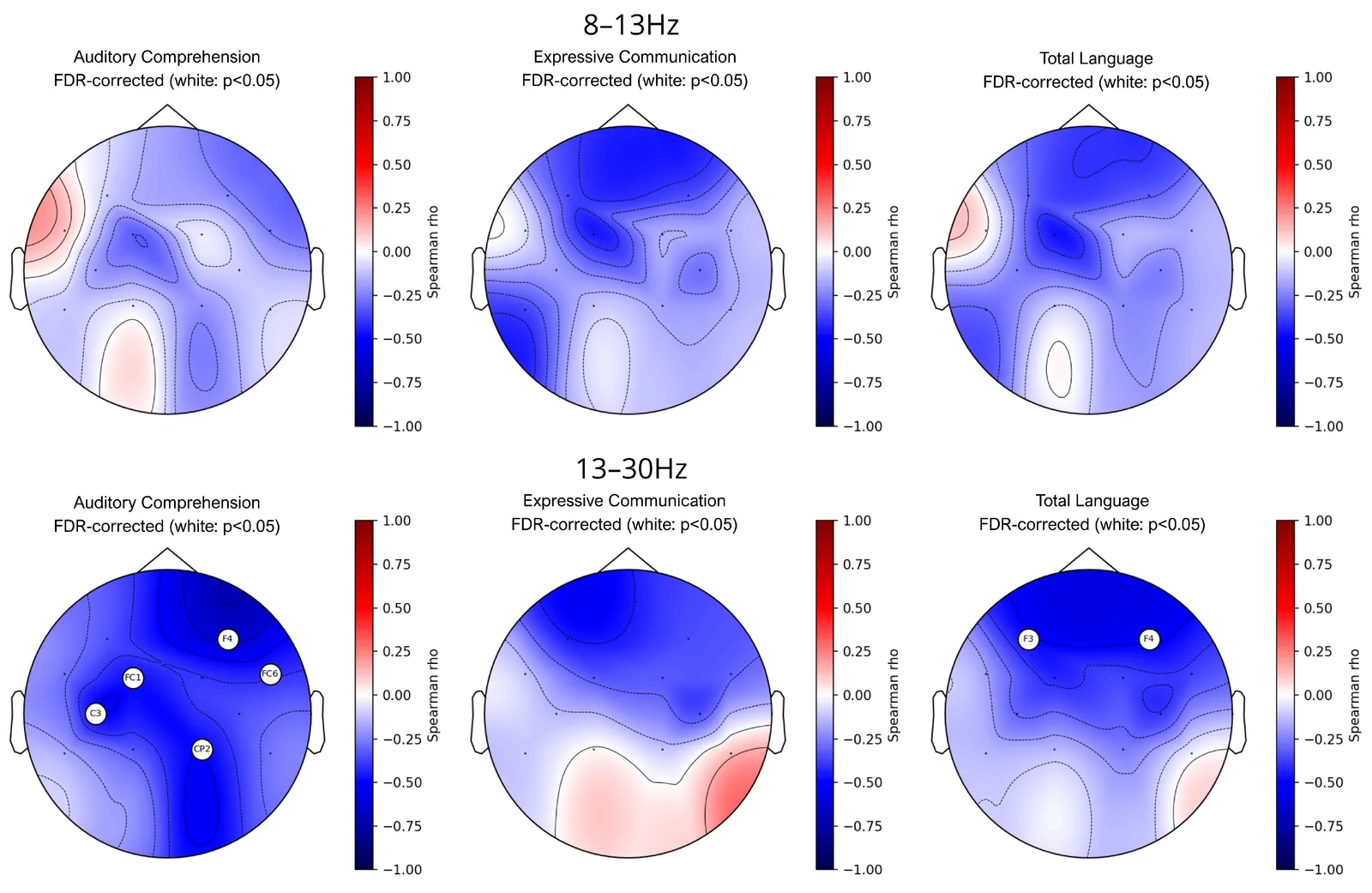
Correlation between the PLS-5 scores (auditory comprehension, expressive communication, total language) and the PHM modulation index for the 8–13 Hz and 13–30 Hz ranges. Channels where significant correlations were found are marked in white.

**Figure 9:**
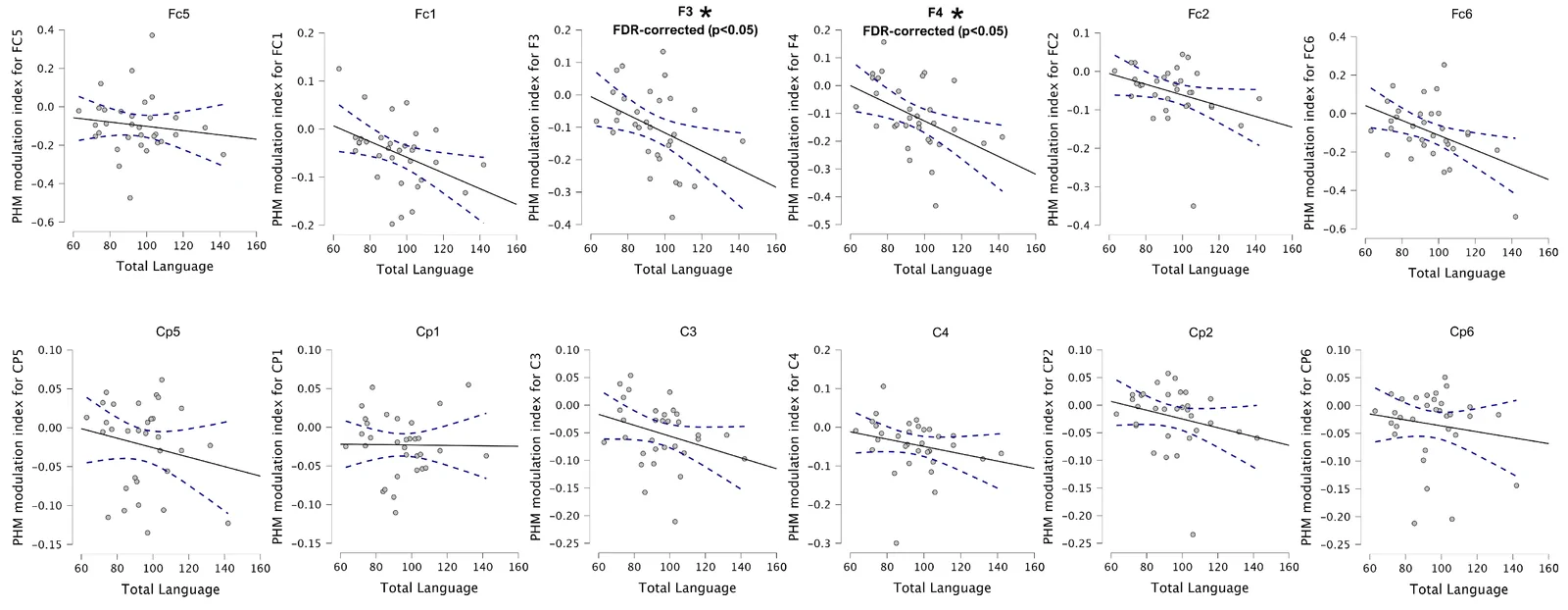
Correlation between the total language and the PHM modulation index for the 13−30 Hz. Grey dots represent individual participants; dashed blue lines represent the 95% confidence interval. Significant correlations are marked “*” and labeled “FDR-corrected (*p* < 0.05)”.

**Figure 10:**
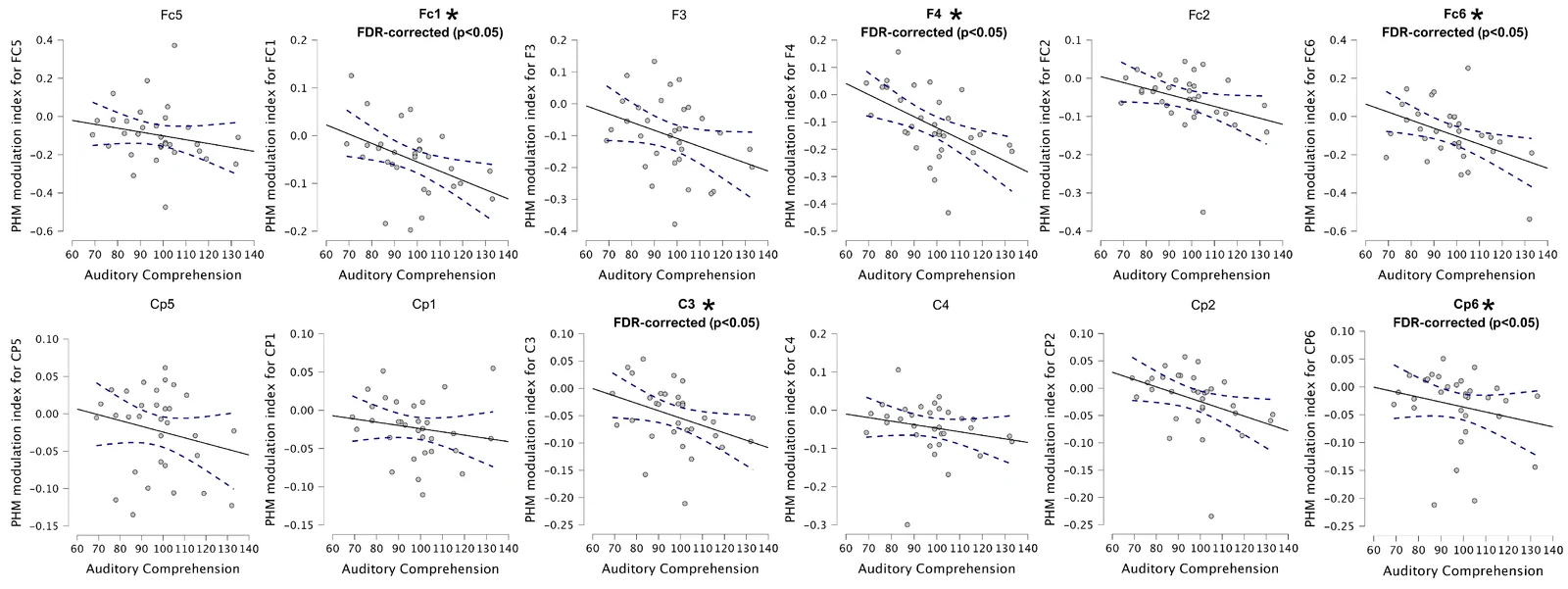
Correlation between the auditory comprehension and the PHM modulation index for the 13−30 Hz. Grey dots represent individual participants; dashed blue lines represent the 95% confidence interval. Significant correlations are marked “*” and labeled “FDR-corrected (*p* < 0.05)”.

Spearman correlation analysis between language development scores (Auditory Comprehension, Expressive Communication, Total Language) and the PHM modulation index for the 8–13 Hz range revealed no significant correlations (see full correlation PLS-5 × PHM for the 8–13 Hz range in Table S4.1 in Supplementary Materials). However, for the 13–30 Hz range, the following significant negative correlations were found: for the Total Language score, significant inverse correlations were observed in the frontal region in channels F3 (Spearman’s ρ = −0.479, *p*(FDR) = 0.033) and F4 (Spearman’s ρ = −0.513, *p*(FDR) = 0.032) (see Figure 8 and Figure 9). For the Auditory Comprehension score, significant inverse correlations were found in the frontal region (channel F4: Spearman’s ρ = −0.619, *p*(FDR) = 0.001), the frontocentral region (channels FC1: Spearman’s ρ = −0.466, *p*(FDR) = 0.021 and FC6 (Spearman’s ρ = −0.417, *p*(FDR) = 0.041), the central region in (channel C3: Spearman’s ρ = −0.482, *p*(FDR) = 0.02) and the centroparietal region in (channel CP2: Spearman’s ρ = −0.487, *p*(FDR) = 0.02) (see Figure 10). The Expressive Communication score revealed no significant correlations. Visualization of correlations between the PLS-5 scores and PHM for both frequency ranges can be seen in Figure 8 (see full correlation PLS-5 × PHM for the 13–30 Hz range in Table S4.2 in Supplementary Materials).

Thus, for the beta range of the sensorimotor rhythm, a consistent inverse relationship was observed between the sensorimotor rhythm desynchronization index and language development: the more pronounced the mu rhythm suppression, the higher the child’s language development, as measured by the PLS-5 scores. No significant correlations were found between the VHM modulation index and language development scores in either frequency range.

## 4. Discussion

The present study showed that passive hand clenching elicited desynchronization of sensorimotor rhythms in both frequency bands. Conversely, viewing hand movements on video induced synchronization of sensorimotor activity in the alpha range, with no modulation in the beta range. Suppression of activity in the hemisphere contralateral to the passively clenched hand was observed only in the alpha range; in the beta range, no lateralization effects were evident. At the same time, no age-related changes in the modulation of sensorimotor rhythms were revealed for either frequency band or experimental conditions, in either longitudinal or cross-sectional comparison. Lateralization effects also did not vary with age (Table 1).

A key finding of the present study is the relationship between language development and the beta-range desynchronization of sensorimotor rhythm during passive hand movement, highlighting a link between sensorimotor neurophysiology and language skills.

### 4.1. Modulation of Sensorimotor Rhythm in Response to Different Experimental Conditions (PHM and VHM)

#### 4.1.1. Passive Hand Movement (PHM)

In the present study, passive hand movement (PHM) elicited desynchronization of the sensorimotor rhythm in both frequency bands, which is consistent with previous studies that used the same paradigm [13,15] and with the established literature on movement-related desynchronization [1].

A limitation of earlier PHM studies [13,15] is that they pooled data across hands. Our study addresses this by separately examining right- and left-hand clenching. Beyond PHM context, other hand movement studies in children aged 3 to 10 years either did not examine right hemisphere activity [5], used only right-hand movement [5,7,9], or did not specify which hand was used [14,26]. In the alpha range, significant mu rhythm desynchronization was observed in the hemisphere contralateral to the hand being clenched, which is consistent with data from adults (for a review, see [1]). In the hemisphere ipsilateral to the clenched hand, no significant suppression compared to the control condition was found for either the right or left hand. Similar results were obtained in a study of right-hand movements in children of this age [7]. Interestingly, interhemispheric asymmetry was more pronounced during right-hand than during left-hand clenching: a lower amplitude in the left hemisphere compared to the right hemisphere was also found in the right-hand movement, whereas during left-hand clenching no analogous interhemispheric differences were observed. The presence of interhemispheric asymmetry specifically for right-hand clenching may reflect the predominance of right-handedness in our sample (33 out of 44 children). No interhemispheric asymmetry was found in the beta range for either hand clenching. This is consistent with some previous studies in this age group [10] but contradicts the work of Trevarrow [11], where interhemispheric asymmetry was reported in motor tasks. The discrepancy with the study by Trevarrow may be attributable to the different age of the participants (their sample: 9–13 years vs. ours: 3–10 years).

The present study also extended previous work by examining the topography of bioelectrical activity in the PHM paradigm beyond central channels (C3 and C4) [13]. For the alpha component of the sensorimotor rhythm, significant suppression was observed in frontocentral (FC1, FC2), central (C3, C4), and centroparietal (CP1, CP2) channels. The observed topography of sensorimotor rhythm desynchronization is consistent with mu rhythm topography [1,14]. Beta-band suppression was more widespread and observed across all channels included in the analysis. These findings extend previously reported results for this age group, where only central channels (C3, Cz, C4) were analyzed [12]. At the same time, MEG studies have reported a more localized beta activity over the primary motor cortex during single-finger movement [10,11]. The broader topography in our study may be explained by two factors: (1) the higher spatial accuracy of MEG compared to EEG and (2) the larger motor and sensorimotor representation of the whole hand compared to a single finger.

The distinct topographies and lateralization patterns of alpha and beta desynchronization indirectly support the view of different functional properties of these components, where the alpha component primarily reflects sensorimotor function, while the beta component is more closely associated with motor preparation and ongoing motor control [1,23].

#### 4.1.2. Video Hand Movement (VHM)

For the video hand movement (VHM) condition, we observed significant synchronization of the sensorimotor rhythm in the alpha range, with no modulation in the beta range. No interhemispheric asymmetry of synchronization or its dominance in any channel was observed. This replicates our previous study [13] and aligns with the theoretical “gating by inhibition” model proposed by Köster and Meyer, which suggests that alpha synchronization during action observation reflects active inhibition of the motor system, preventing overt movement while observing others, who proposed [33].

EEG studies have consistently demonstrated that observing the actions of others engages a distinct cortical network—the action observation network (AON)—which overlaps with regions activated during actual movement execution. The mu rhythm desynchronization during action observation is widely interpreted as an index of the human mirror neuron system, supporting action understanding, imitation, and social cognition [34]. However, mu suppression during passive observation depends on many experimental and individual factors. The action observation network and the general visual attention system may overlap with one another, making it difficult to disentangle their respective contributions. Mu activity is influenced by such stimulus characteristics as background color, object content, and attentional demands [35]. Thus, the choice of baseline/control condition is crucial [36]. Tangwiriyasakul’s study showed that different adult participants exhibited more pronounced effects of mu rhythm suppression under different baseline conditions [37]. Even with the selected optimal baseline condition, mu rhythm suppression during movement observation was absent in 30% of the participants. Similarly, Hobson 2016 [36] reported that 16–21% of participants did not exhibit suppression, likely due to differences in attention engagement and experimental conditions; for example, the baseline (control) condition may be more appealing to some participants.

The observer’s motor experience also modulates the magnitude of the mirror motor response during observation of an action [38]. For example, in infants, rhythm suppression occurs only when observing movements from their motor repertoire [39]. It is believed that observing a familiar and a new action elicits different cortical connectivity modulations in the alpha and beta ranges [40]. Using TMS-EEG co-registration in adults, Guidali and colleagues showed that typical, naturally acquired motor resonance during action observation was linked to prominent alpha-band connectivity of the primary motor cortex, whereas beta-band connectivity was more selectively associated with newly acquired sensorimotor associations [40]. Therefore, it is plausible that the alpha-band synchronization observed during VHM in our study may reflect the engagement of an already established action-observation mechanism, consistent with the absence of any beta-band modulation in this condition.

In summary, both previous studies and our research indicate that the action observation paradigm requires further thorough investigation to clarify the conditions under which synchronization versus desynchronization occurs, and to disentangle the distinct contribution of the alpha and beta components of sensorimotor rhythms in this context.

### 4.2. Age-Related Changes in Sensorimotor Rhythm

#### 4.2.1. Changes in Sensorimotor Rhythms over the Course of One Year

Within the framework of a mixed longitudinal study of sensorimotor rhythm modulation, we hypothesized that longitudinal changes in mu rhythm modulation would be observed in children over the course of one year. However, this research hypothesis was not confirmed. The amplitude in both the alpha and beta frequency ranges changed over the one-year period independently of experimental conditions. This finding, first and foremost, indicates active maturation of the brain’s bioelectrical activity in children aged 3 to 10 years, which is consistent with the general developmental patterns of rhythmic activity [5,19]. At the same time, the modulation of sensorimotor rhythms under PHM and VHM conditions was already present from the age of 3 years, yet remained stable over the course of one year.

#### 4.2.2. Cross-Sectional Changes in Sensorimotor Rhythms in Children 3–10 Years of Age

##### Alpha Component of the Sensorimotor Rhythm

In our study, the modulation of sensorimotor rhythm in the alpha range by either passive hand movement or hand movement observation was stable across the studied period from 3 to 10 years of age in typically developing children, as confirmed by the absence of interaction of factor Condition with either Age or Longitudinal Time Point. Thus, no cross-sectional changes in sensorimotor rhythm modulation were observed in the alpha range across the studied age period of 3 to 10 years. This finding is generally in line with previous studies of children of a similar age range.

In the studies by Eismont (sample aged 4 to 14 years) and Lepage (sample aged 6 to 11 years), there was no age-related modulation either during the observation of movement or during its execution [7,41]. At the same time, Thorpe identified age-related changes in the modulation of sensorimotor rhythms during active movement execution [14]. A similar pattern of age-related increase in alpha desynchronization was observed for the passive hand movement condition (PHM) by the study of Mitiureva, from whom we adopted this experimental paradigm [15]. However, such an increase might be attributed to the wide age range of the samples that included children and adults. At the same time, in the movement observation condition of the Mitiureva study (sample aged 4 to 31 years), no age-related modulation was detected, whereas in the studies by Oberman (sample aged 6 to 47 years) and Brunsdon (sample aged 10 to 86 years), modulation increased with age [42,43]. It may be assumed that detecting age-related changes in modulation requires a broader age range; it is possible that more pronounced changes in modulation occur after the age of 10.

It should be noted that the research paradigm used can also affect age modulation. So, most of the studies mentioned above [7,14,41] used self-initiated movement, requiring participants to plan and exert voluntary control. The PHM paradigm in our study was conducted by a member of the research team and did not require any action on the part of the participant. Studies in adults have shown that the degree of desynchronization of the sensorimotor rhythm during movement execution may depend on the complexity of the movement [1]. It can therefore be assumed that age-related changes in desynchronization may be modulated by the degree of involvement of motor control processes, which continue to mature throughout childhood. To clarify this issue, further research is needed comparing the modulation of the sensorimotor rhythm in tasks with varying levels of involvement and movement complexity.

##### Beta Component of the Sensorimotor Rhythm

The beta-band desynchronization during passive hand clenching expanded with age in the topography, extending from frontocentral regions in children aged 3 to 4 years to all regions included in the analysis by the age of 7–8 years. MEG study of movement-related event-related desynchronization during finger movements comparing two groups of children aged 4 to 6 and 11 to 13 years, as well as a group of adults aged 24 to 42 years, reported an increase in movement-related beta rhythm desynchronization [10]. However, the authors did not exclude the possibility that the increase in signal strength may be related to the reduction in the distance between the MEG sensors and the cortex as head size increases. A later similar MEG study of children aged 9 to 15 years found no age-related changes in sensorimotor rhythm modulation [11]. In Mitiureva’s study, age-related modulation of beta desynchronization was observed, but, as mentioned above, within a larger age range of 4–31 years old. Taken together, these findings suggest that, similarly to the alpha component of the mu rhythm, detecting age-related changes in beta component modulation may require a broader age range than that represented in the present study. Moreover, it cannot be excluded that more pronounced age-related modulation of beta sensorimotor activity may emerge after the age of 10–11 years.

##### Age-Related Changes in the Lateralization of Sensorimotor Activity During Right- and Left-Passive Hand Movement

Initially, we hypothesized that the lateralization of sensorimotor activity would increase with age in our sample of 3–10-year-old children. This hypothesis was based on the behavioral study by Scharoun and Bryden that demonstrates an establishment of a pronounced hand preference, especially in right-handers, only by age 6, with children aged 3 to 5 years showing only weak and inconsistent tendencies [17]. However, the results of our study revealed no age-related changes in interhemispheric differences during passive hand clenching of the right or left hand from age 3 onward. These discrepancies between neurophysiological lateralization of mu rhythm and behavioral handedness may be due to two factors. First, our paradigm did not require voluntary control of one’s own movement, which may be crucial for observing age-related increases in lateralization. Second, our sample size may have been insufficient to detect subtle developmental effects, should they exist. Future studies using active movement paradigms and larger samples are needed to clarify this issue.

### 4.3. Relationship Between Sensorimotor Rhythm and Language Development

In the present study, we found a negative correlation between the PLS-5 language measures (Total Language and Auditory Comprehension) and the magnitude of beta-band desynchronization during passive hand movement. Notably, a fairly extensive region of negative correlation with Auditory Comprehension was observed (spanning channels F4, FC1, FC6, C3 and CP2), whereas no such correlations were found with Expressive Communication. This result is unexpected, as a relationship between expressive (motor-related) language skills and the sensorimotor rhythm would have been more anticipated. However, our results suggest that receptive language, rather than production, relies more heavily on the recruitment of extensive sensorimotor networks in this age group. This suggests that the sensorimotor system may play a supportive role in decoding auditory-verbal information, even when no overt motor output is required. To the best of our knowledge, our study is the first to describe the relationship between the sensorimotor system and language development in preschoolers and early school-age children aged 3 to 10 years. Although limited in number, previous studies on the contribution of the sensorimotor network to language development, and to auditory language comprehension in particular, have focused mainly on infants and children in early childhood up to 3–4 years of age (for a review, see [27,44,45]).

A relationship between sensorimotor rhythm development and Auditory Comprehension scores (assessed with the Bayley Scales of Infant and Toddler Development, Third Edition, BSID-III) was found in the study by Mikhailova, but for a younger age group (1.5–3.5 years; mean age 2.5 years) and for the alpha component of the mu rhythm (the beta component was not examined), in the left frontal and parietal channels (F3 and P3) [26]. The alpha and beta components of the sensorimotor rhythms in studies on the role of the sensorimotor system in speech perception in children have previously been discussed only in the work of Antognini and Daum [46]. That study examined how the presentation of a verb that was either congruent or incongruent with a subsequent action influenced action perception in children aged 23 to 24 months. According to their results, children with higher language skills exhibited suppression of the sensorimotor rhythm in the beta range, whereas children with lower language skills (a smaller verb vocabulary) showed suppression in the alpha range when viewing congruent stimuli (when the action matched the preceding verb). Similar results, but only in the alpha range, were obtained by Patzwald with a similar study design in younger children aged 18 months: children with better language skills showed greater mu rhythm desynchronization than children with lower language skills [25]. Thus, it can be suggested that with age, beta activity plays an increasingly important role in language comprehension, which is consistent with the results of our study.

In preverbal infants, discrimination of speech sounds is closely linked to the articulation of those sounds; inhibiting articulation leads to a selective impairment in the ability to discriminate sounds associated with that particular articulation, while not affecting the differentiation of other speech sounds [44]. Additionally, the sensorimotor network may play a supportive role in language development in infants through the coupling of gestural and verbal communication [45]. Ohashi and Ostry found that different brain regions are engaged during language learning in children and adults: in adults, the associative auditory cortex was involved, whereas in children aged 5 to 12 years, somatosensory areas and primary motor cortex were activated [47]. The role of the sensorimotor system in speech perception in adults was most comprehensively presented in the review by Skipper, who identified an extensive overlap of neural areas functionally involved in speech perception and production; areas involved in speech production are ubiquitously and specifically recruited during speech perception, with the degree of their activation being dynamically modulated by context, for example, increasing under difficult acoustic conditions [48]. All these data support a link between the sensorimotor network and speech and language development; however, further research is needed to investigate the associations between somatosensory processes and the development of Auditory Comprehension and Expressive Communication, including comprehensive studies of sensorimotor rhythms during speech listening, articulation, and speaking.

Thus, we confirm the study hypothesis that sensorimotor rhythm is associated with language development in preschool and early school-age children. If mu rhythm suppression is considered a neurophysiological marker of sensorimotor system development, then children with a more mature sensorimotor system demonstrate more advanced language abilities.

## 5. Conclusions

This mixed longitudinal study examined age-related changes in sensorimotor (mu) rhythm modulation and its relationship with language development in children aged 3 to 10 years.

First, contrary to our hypothesis, we found no age-related changes in modulation strength for either the alpha or beta component of sensorimotor rhythm, in either the passive hand movement or hand movement observation. Modulation was already present at age 3 and remained stable across the studied period, both cross-sectionally and over one year. Similarly, the research hypothesis that the lateralization of sensorimotor activity during right- or left- passive hand movements increases with age was not confirmed. While alpha desynchronization was strictly contralateral to the moved hand, this pattern did not strengthen with age, suggesting that the basic neurophysiological asymmetry of sensorimotor responses is established early in development, at least for passive movements.

Most importantly, the study identified a relationship between beta-band desynchronization during passive hand movement and language scores on the PLS-5, supporting the third study hypothesis that sensorimotor rhythms are associated with language development. Notably, correlations with Auditory Comprehension were observed across a fairly extensive region, whereas no correlation was found for Expressive Communication. This suggests that receptive language relies more heavily on sensorimotor network engagement than expressive language at this age group.

These findings provide novel evidence linking beta-band sensorimotor desynchronization to receptive language development in preschool and early school-age children, suggesting a potential neurophysiological marker of individual differences in language ability. Given the clinical relevance of early language acquisition, these results may contribute to the development of screening tools for children at risk for language delays, though replication in larger samples is needed.

## 6. Limitations

Our null finding regarding the age-related effect on sensorimotor rhythm modulation should be interpreted with caution, as our sample and analytical approach have limited ability to detect small effects. For example, several borderline effects of interest emerged, as can be seen in the Supplementary Tables: the Longitudinal TP × Condition × Region × Age Group interaction for the alpha component of the sensorimotor rhythm (*p* = 0.051, *η*^2^*p* = 0.072); the Longitudinal Time Point × Condition interaction for the beta component (*p* = 0.067, *η*^2^*p* = 0.064); the cross-sectional Condition × Age Group interaction for the beta component (*p* = 0.063, *η*^2^*p* = 0.102); and the Lateralization × Region × Age Group interaction for beta component lateralization (*p* = 0.067, *η*^2^*p* = 0.069). A sensitivity power analysis conducted in G*Power 3.1 indicated that the minimum detectable effect sizes (power = 80%, α = 0.05) are *η*^2^*p* = 0.103, *η*^2^*p* = 0.073, *η*^2^*p* = 0.132, and *η*^2^*p* = 0.113, respectively, for those analyses, which are larger than the observed effect sizes, pointing to insufficient statistical power to detect effects of the observed magnitude. Future research should incorporate larger samples to be able to detect such small effects, while we only claim the absence of medium and large age-related effects within the studied age range (3–9 years of age).

In addition, several further limitations should be noted. The age groups were unbalanced in terms of the number of children, which may have reduced the statistical power of between-group comparisons and increased the risk of Type II errors. Only 32 of the 44 participants completed the PLS-5, which was used to assess language development at the first longitudinal time point. In addition, language development was not assessed at the second longitudinal time point, which would have made it possible to compare the dynamics of language development with the dynamics of changes in sensorimotor rhythms. Lateralization effects were not examined at the first longitudinal time point; thus, changes in lateralization with age at the individual level remained unexplored. Finally, the duration of the VFM and VHM paradigms changed over the course of the first longitudinal time point, from 30 s to 60 s, and later to 90 s. We did not standardize the duration of the VFM and VHM paradigms to a single value for analysis; this variability resulted in a different number of available EEG epochs across participants. Longer recordings tend to yield more balanced average signal amplitude values, but are more susceptible to decreased attention toward the end of the recording, whereas shorter segments are less prone to artifacts and outliers. Thus, we cannot exclude the possibility that the varying paradigm duration at the first longitudinal time point may have introduced some degree of distortion into the obtained results, and future studies should aim for greater unification and standardization of experimental paradigms.

## Figures and Tables

**Table 1:** Summary of main condition-related findings across the alpha (8–13 Hz) and beta (13–30 Hz) sensorimotor rhythm components, including region topography, lateralization, cross-sectional and longitudinal age-related effects, and correlations with language development (PLS-5) scores. PHM = passive hand movement; VHM = video hand movement observation; desync. = desynchronization; sync. = synchronization.

Effect	Alpha (8–13 Hz)	Beta (13–30 Hz)
Modulation by Condition	PHM: desync.	PHM: desync.
	VHM: sync.	VHM: not significant
Region topography of differences with control (VFM)	PHM: FC1/FC2, C3/C4, CP1/CP2	PHM: F3/F4, FC5/FC6, FC1/FC2, C3/C4, CP5/CP6, CP1/CP2
	VHM: not significant	VHM: not significant
Lateralization	PHM: contralateral (stable with age)	not significant *
	VHM: not significant	
Cross-sectional (Condition × Age Group)	not significant *	not significant *
Longitudinal (Condition × Time Point)	not significant *	not significant *
Language correlation (PLS-5)	not significant *	PHM desync. negativ. correl. with Auditory Comprehension
		VHM: not significant

Not significant * = not significant across all conditions.

## Data Availability

The scripts and data used for this study can be found at the following link: https://osf.io/mnuaz/overview (accessed on 13 August 2026).

## Supplementary Materials

The following supporting information can be downloaded at https://www.mdpi.com/article/10.3390/brainsci16080880/s1, Table S1: RM Anova for 8–13 Hz Range; Table S1.2: Post Hoc Comparisons—Condition for main RM Anova 8–13 Hz Range; Table S1.3: Post Hoc Comparisons—Condition × Region for main RM Anova 8–13 Hz Range; Figure S1: Post hoc for the Condition × Region interaction in the 8–13 Hz range revealed significant differences between the VFM vs PHM conditions, as well as between the PHM vs VHM conditions, in the fronto-central (FC1/FC2), central (C3/C4), and centro-parietal (CP1/CP2) regions. Significant differences are marked with brackets and “*”; Table S2: RM Anova for 13–30 Hz Range; Table S2.2: Post Hoc Comparisons—Condition for main RM Anova 13–30 Hz Range; Table S2.3: Post Hoc Comparisons—Condition × Region for main RM Anova 13–30 Hz Range; Table S2.4: Post Hoc Comparisons—Age group for main RM Anova 13–30 Hz Range; Table S2.5.1: RM Anova for the 3–4 years-old in the 13–30 Hz Range; Table S2.5.1.2: Post Hoc Comparisons—Condition for RM Anova for the 3–4 years-old in the 13–30 Hz Range; Table S2.5.1.3: Post Hoc Comparisons—Condition × Region for RM Anova for the 3–4 years-old in the 13–30 Hz Range; Table S2.5.2: RM Anova for the 5–6 years-old in the 13–30 Hz Range; Table S2.5.2.2: Post Hoc Comparisons—Condition for RM Anova for the 5–6 years-old in the 13–30 Hz Range; Table S2.5.2.3: Post Hoc Comparisons—Condition × Region for RM Anova for the 5–6 years-old in the 13–30 Hz Range; Table S2.5.3: RM Anova for the 7–8 years-old in the 13–30 Hz Range; Table S2.5.3.2: Post Hoc Comparisons—Condition for RM Anova for the 7–8 years-old in the 13–30 Hz Range; Figure S2: Mean amplitude spectra in the 8–13 Hz range at channels C3 and C4, shown separately for each age subgroup (3–4, 5–6, 7–8 years) and experimental condition (PHM right hand, PHM left hand, VFM, VHM). Spectra are averaged across all participants within each age subgroup at the first longitudinal time point.; Figure S3: Mean amplitude spectra in the 13–30 Hz range at channels C3 and C4, shown separately for each age subgroup (3–4, 5–6, 7–8 years) and experimental condition (PHM right hand, PHM left hand, VFM, VHM). Spectra are averaged across all participants within each age subgroup at the first longitudinal time point.; Table S3.1: RM Anova of Lateralization Effects during Right- and Left-Hand clenching in the 8–13 Hz Range; Table S3.1.2: Post Hoc Comparisons—Lateralization for RM Anova Lateralization Effects in the 8–13 Hz Range; Table S3.1.3: Post Hoc Comparisons—Lateralization × Hemisphere for RM Anova Lateralization Effects in the 8–13 Hz Range; Table S3.1.4a: Post Hoc Comparisons—Lateralization × Hemisphere × Region for RM Anova Lateralization Effects in the 8–13 Hz Range. Different experimental conditions within the same hemisphere and region; Table S3.1.4b: Post Hoc Comparisons—Lateralization × Hemisphere × Region for RM ANOVA Lateralization Effects in the 8–13 Hz Range. Different hemispheres within the same experimental condition and region; Table S3.1.5: Post Hoc Comparisons—Age group for RM Anova Lateralization Effects in the 8–13 Hz Range; Table S3.2: RM Anova of Lateralization Effects during Right- and Left-Hand clenching in the 13–30 Hz Range; Table S3.2.2: Post Hoc Comparisons—Lateralization for RM Anova Lateralization Effects in the 13–30 Hz Range; Table S3.2.3: Post Hoc Comparisons—Age group for RM Anova Lateralization Effects in the 13–30 Hz Range; Table S4.1: Correlation between PLS-5 scores and Modulation Index PHM in the 8–13 Hz range; Table S4.2: Correlation between PLS-5 scores and Modulation Index PHM in the 13–30 Hz range.

## Abbreviations

The following abbreviations are used in this manuscript:

VHM	Video Hand Movement
VFM	Video Fractal Movement
PHM	Passive Hand Movement
EEG	electroencephalography
ICA	Independent Component Analysis
CSD	Current Source Density
FFT	Fast Fourier Transform
RM ANOVA	Repeated Measures Analysis of Variance
