# Supplementary material for: A Mixed Longitudinal EEG Study of Sensorimotor Rhythm Modulation and Its Relationship with Language Development in Children Aged 3–10 Years
Source: Brain Sci. 2026 Aug 18;16(8):880. doi: 10.3390/brainsci16080880 (PMC13511290; doi:10.3390/brainsci16080880)
Supplement: Supplementary file 1 [file brainsci-16-00880-s001.zip › brainsci-4468871-supplementary.pdf]

Longitudinal EEG Study of Sensorimotor Rhythm Modulation in Children Aged 3–10 Years and Its Association with Speech Development

Supplementary Materials

Sensorimotor rhythms characteristics for 8–13Hz Range

Table S1: RM Anova for 8–13Hz Range

Within Subjects Effects						
Cases	Sum of Squares	df	Mean Square	F	p	η²p
**Longitudinal TP**	**5.861×10-6**	**1**	**5.861×10-6**	**27.85**	**< 0.001**	**0.405**
Longitudinal TP × Age group	1.503×10-7	2	7.514×10-8	0.357	0.702	0.017
**Condition**	**6.786×10-6**[a]	**2**[a]	**3.393×10-6**[a]	**24.49**[a]	**< 0.001**[a]	**0.374**
Condition × Age group	3.922×10-7[a]	4[a]	9.805×10-8[a]	0.708[a]	0.589[a]	0.033
Hemisphere	2.233×10-7	1	2.233×10-7	1.909	0.175	0.044
Hemisphere × Age group	5.466×10-9	2	2.733×10-9	0.023	0.977	0.001
**Region**	**3.255×10-5**[a]	**5**[a]	**6.511×10-6**[a]	**44.50**[a]	**< 0.001**[a]	**0.520**
Region × Age group	1.273×10-6[a]	10[a]	1.273×10-7[a]	0.870[a]	0.562[a]	0.041
Longitudinal TP × Condition	2.012×10-8	2	1.006×10-8	0.147	0.863	0.004
Longitudinal TP × Condition ×Age group	3.989×10-7	4	9.972×10-8	1.458	0.223	0.066
Condition × hemisphere	4.026×10-8	2	2.013×10-8	0.856	0.428	0.020
Condition × hemisphere × Age group	1.840×10-8	4	4.601×10-9	0.196	0.940	0.009
**Condition × Region**	**3.107×10-6**[a]	**10**[a]	**3.107×10-7**[a]	**9.785**[a]	**< 0.001**[a]	**0.193**
Condition × Region × Age group	6.454×10-7[a]	20[a]	3.227×10-8[a]	1.016[a]	0.441[a]	0.047
Longitudinal TP × Condition ×hemisphere	3.676×10-9	2	1.838×10-9	0.107	0.899	0.003
Longitudinal TP × Condition ×hemisphere × Age group	1.627×10-8	4	4.067×10-9	0.236	0.917	0.011
Longitudinal TP × Condition ×Region	1.877×10-7[a]	10[a]	1.877×10-8[a]	1.086[a]	0.371[a]	0.026
Longitudinal TP ×	5.498×10-7[a]	20[a]	2.749×10-8[a]	1.591[a]	0.051[a]	0.072

Condition ×Region × Age group						
Condition × hemisphere × Region	1.065×10-7[a]	10[a]	1.065×10-8[a]	0.912[a]	0.522[a]	0.022
Condition × hemisphere × Region ×Age group	1.520×10-7[a]	20[a]	7.599×10-9[a]	0.651[a]	0.873[a]	0.031
Longitudinal TP × Condition ×hemisphere × Region	7.991×10-8[a]	10[a]	7.991×10-9[a]	0.811[a]	0.618[a]	0.019
Longitudinal TP × Condition ×hemisphere × Region × Age group	1.097×10-7[a]	20[a]	5.483×10-9[a]	0.557[a]	0.940[a]	0.026
Within Subjects Effects						
Cases	Sum of Squares	df	Mean Square	F	p	η^2p
Age group	2.171×10-5	2	1.086×10-5	3.138	0.054	0.133

Table S1.2: Post Hoc Comparisons—Condition for main RM Anova 8–13 Hz Range.

Post Hoc Comparisons - Condition							
		Mean Difference	SE	df	t	Cohen's d	pHolm
**VFM**	**PHM**	**8.461×10-5**	**1.449×10-5**	**41**	**5.840**	**0.285**	**< 0.001**
**VFM**	**VHM**	**-3.640×10-5**	**1.695×10-5**	**41**	**-2.148**	**-0.123**	**0.038**
**PHM**	**VHM**	**-1.210×10-4**	**2.115×10-5**	**41**	**-5.721**	**-0.407**	**< 0.001**

Table S1.3: Post Hoc Comparisons—Condition × Region for main RM Anova 8–13 Hz Range

Post Hoc Comparisons - Condition × Region							
		Mean Difference	SE	df	t	Cohen's d	pHolm
VFM, F(3/4)	PHM, F(3/4)	5.883×10-5	2.453×10-5	41	2.398	0.198	0.739
VFM, F(3/4)	VHM, F(3/4)	-6.269×10-7	2.358×10-5	41	-0.027	-0.002	1.000
PHM, F(3/4)	VHM, F(3/4)	-5.946×10-5	2.685×10-5	41	-2.214	-0.200	1.000
VFM, Fc(5/6)	PHM, Fc(5/6)	5.049×10-5	2.320×10-5	41	2.176	0.170	1.000
VFM, Fc(5/6)	VHM, Fc(5/6)	-3.410×10-5	2.311×10-5	41	-1.476	-0.115	1.000
PHM, Fc(5/6)	VHM, Fc(5/6)	-8.459×10-5	2.622×10-5	41	-3.227	-0.285	0.130
**VFM, Fc(1/2)**	**PHM, Fc(1/2)**	**6.889×10-5**	**1.568×10-5**	**41**	**4.395**	**0.232**	**0.006**
VFM, Fc(1/2)	VHM, Fc(1/2)	-4.690×10-5	1.695×10-5	41	-2.767	-0.158	0.380
**PHM, Fc(1/2)**	**VHM, Fc(1/2)**	**-1.158×10-4**	**2.071×10-5**	**41**	**-5.591**	**-0.390**	**< 0.001**
**VFM, C(3/4)**	**PHM, C(3/4)**	**2.017×10-4**	**3.450×10-5**	**41**	**5.846**	**0.679**	**< 0.001**
VFM, C(3/4)	VHM, C(3/4)	-9.695×10-5	4.619×10-5	41	-2.099	-0.326	1.000

**PHM, C(3/4)**	**VHM, C(3/4)**	**-2.986×10-4**	**4.848×10-5**	**41**	**-6.160**	**-1.006**	**< 0.001**
VFM, Cp(5/6)	PHM, Cp(5/6)	5.188×10-5	1.555×10-5	41	3.335	0.175	0.107
VFM, Cp(5/6)	VHM, Cp(5/6)	-7.261×10-6	1.726×10-5	41	-0.421	-0.024	1.000
PHM, Cp(5/6)	VHM, Cp(5/6)	-5.914×10-5	2.317×10-5	41	-2.552	-0.199	0.552
**VFM, Cp(1/2)**	**PHM, Cp(1/2)**	**7.588×10-5**	**1.494×10-5**	**41**	**5.080**	**0.255**	**< 0.001**
VFM, Cp(1/2)	VHM, Cp(1/2)	-3.259×10-5	1.487×10-5	41	-2.192	-0.110	1.000
**PHM, Cp(1/2)**	**VHM, Cp(1/2)**	**-1.085×10-4**	**1.910×10-5**	**41**	**-5.680**	**-0.365**	**< 0.001**

Figure S1: Post hoc for the Condition × Region interaction in the 8–13 Hz range revealed significant differences between the VFM vs PHM conditions, as well as between the PHM vs VHM conditions, in the fronto-central (FC1/FC2), central (C3/C4), and centro-parietal (CP1/CP2) regions. Significant differences are marked with brackets and "*".

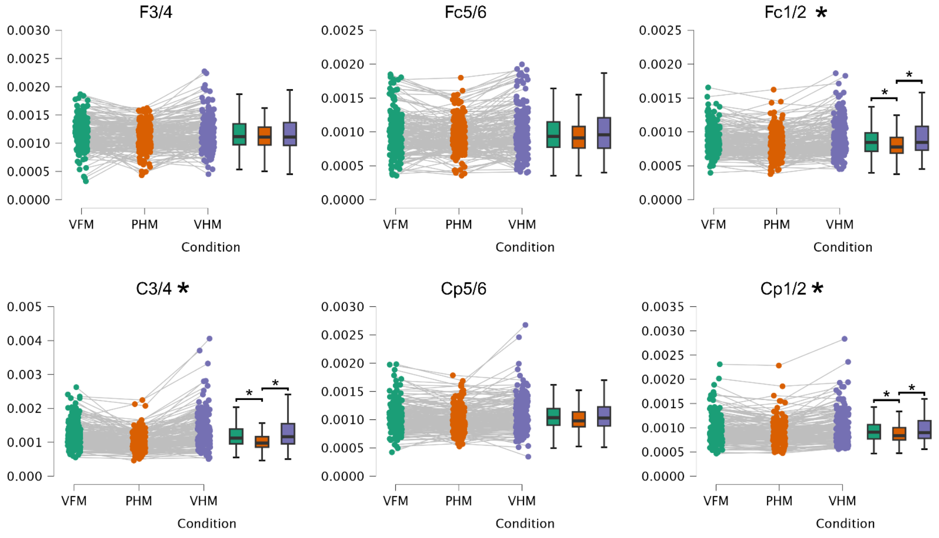

Sensorimotor rhythms characteristics for 13–30Hz Range

Table S2: RM Anova for 13–30 Hz Range.

Within Subjects Effects						
Cases	Sum of Squares	df	Mean Square	F	p	η²p
**Longitudinal TP**	**5.438×10-6**	**1**	**5.438×10-6**	**50.14**	**< 0.001**	**0.550**
Longitudinal TP× Age group	5.006×10-8	2	2.503×10-8	0.231	0.795	0.011
**Condition**	**4.805×10-6**	**2**	**2.402×10-6**	**35.82**	**< 0.001**	**0.466**
Condition × Age group	6.253×10-7	4	1.563×10-7	2.331	0.063	0.102
**Hemisphere**	**1.483×10-7**	**1**	**1.483×10-7**	**4.182**	**0.047**	**0.093**
Hemisphere × Age group	3.366×10-8	2	1.683×10-8	0.475	0.626	0.023
**Region**	**6.514×10-5[a]**	**5[a]**	**1.303×10-5[a]**	**170.9[a]**	**< 0.001[a]**	**0.807**

**Region × Age group**	**4.586×10-6[a]**	**10[a]**	**4.586×10-7[a]**	**6.015[a]**	**< 0.001[a]**	**0.227**
Longitudinal TP × Condition	3.243×10-7[a]	2[a]	1.621×10-7[a]	2.797[a]	0.067[a]	0.064
Longitudinal TP × Condition ×Age group	2.888×10-7[a]	4[a]	7.221×10-8[a]	1.246[a]	0.298[a]	0.057
Condition × Hemisphere	1.368×10-8[a]	2[a]	6.841×10-9[a]	0.662[a]	0.519[a]	0.016
Condition × Hemisphere × Age group	1.804×10-8[a]	4[a]	4.509×10-9[a]	0.436[a]	0.782[a]	0.021
**Condition × Region**	**3.211×10-6[a]**	**10[a]**	**3.211×10-7[a]**	**12.72[a]**	**< 0.001[a]**	**0.237**
**Condition × Region × Age group**	**9.417×10-7[a]**	**20[a]**	**4.708×10-8[a]**	**1.865[a]**	**0.014[a]**	**0.083**
Longitudinal TP × Condition ×Hemisphere	3.574×10-8	2	1.787×10-8	1.197	0.307	0.028
Longitudinal TP × Condition ×Hemisphere × Age group	2.388×10-8	4	5.971×10-9	0.400	0.808	0.019
Longitudinal TP × Condition ×Region	3.099×10-7[a]	10[a]	3.099×10-8[a]	1.651[a]	0.090[a]	0.039
Longitudinal TP × Condition ×Region × Age group	3.737×10-7[a]	20[a]	1.869×10-8[a]	0.995[a]	0.467[a]	0.046
Condition × Hemisphere × Region	2.445×10-8[a]	10[a]	2.445×10-9[a]	0.278[a]	0.986[a]	0.007
Condition × Hemisphere × Region ×Age group	1.099×10-7[a]	20[a]	5.497×10-9[a]	0.625[a]	0.894[a]	0.030
Longitudinal TP × Condition ×Hemisphere × Region	5.073×10-8[a]	10[a]	5.073×10-9[a]	0.584[a]	0.828[a]	0.014
Longitudinal TP × Condition ×Hemisphere × Region × Age group	1.986×10-7[a]	20[a]	9.929×10-9[a]	1.142[a]	0.303[a]	0.053
Within Subjects Effects						
Cases	Sum of Squares	df	Mean Square	F	p	$\eta^2 p$
**Age group**	**6.890×10-6**	**2**	**3.445×10-6**	**4.746**	**0.014**	**0.188**

Table S2.2: Post Hoc Comparisons—Condition for main RM Anova 13–30 Hz Range.

Post Hoc Comparisons - Condition							
		Mean Difference	SE	df	t	Cohen's d	pHolm
**VFM**	**PHM**	**9.257×10-5**	**1.056×10-5**	**41**	**8.767**	**0.491**	**< 0.001**

VFM	VHM	4.332×10-6	1.328×10-5	41	0.326	0.023	0.746
**PHM**	**VHM**	**-8.824×10-5**	**1.301×10-5**	**41**	**-6.781**	**-0.468**	**< 0.001**

Table S2.3: Post Hoc Comparisons—Condition × Region for main RM Anova 13–30 Hz Range

Post Hoc Comparisons - Condition × Region							
		Mean Difference	SE	df	t	Cohen's d	pHolm
**VFM, F(3/4)**	**PHM, F(3/4)**	**2.118×10-4**	**3.525×10-5**	**41**	**6.009**	**1.123**	**< 0.001**
VFM, F(3/4)	VHM, F(3/4)	1.384×10-5	3.793×10-5	41	0.365	0.073	1.000
**PHM, F(3/4)**	**VHM, F(3/4)**	**-1.980×10-4**	**3.721×10-5**	**41**	**-5.320**	**-1.050**	**< 0.001**
**VFM, Fc(5/6)**	**PHM, Fc(5/6)**	**1.708×10-4**	**2.642×10-5**	**41**	**6.464**	**0.906**	**< 0.001**
VFM, Fc(5/6)	VHM, Fc(5/6)	-1.702×10-5	3.427×10-5	41	-0.497	-0.090	1.000
**PHM, Fc(5/6)**	**VHM, Fc(5/6)**	**-1.878×10-4**	**3.350×10-5**	**41**	**-5.607**	**-0.996**	**< 0.001**
**VFM, Fc(1/2)**	**PHM, Fc(1/2)**	**4.892×10-5**	**8.873×10-6**	**41**	**5.513**	**0.259**	**< 0.001**
VFM, Fc(1/2)	VHM, Fc(1/2)	4.649×10-6	8.997×10-6	41	0.517	0.025	1.000
**PHM, Fc(1/2)**	**VHM, Fc(1/2)**	**-4.427×10-5**	**8.754×10-6**	**41**	**-5.057**	**-0.235**	**< 0.001**
**VFM, C(3/4)**	**PHM, C(3/4)**	**6.484×10-5**	**8.851×10-6**	**41**	**7.326**	**0.344**	**< 0.001**
VFM, C(3/4)	VHM, C(3/4)	9.471×10-6	1.121×10-5	41	0.845	0.050	1.000
**PHM, C(3/4)**	**VHM, C(3/4)**	**-5.537×10-5**	**9.892×10-6**	**41**	**-5.598**	**-0.294**	**< 0.001**
**VFM, Cp(5/6)**	**PHM, Cp(5/6)**	**3.757×10-5**	**8.953×10-6**	**41**	**4.196**	**0.199**	**0.004**
VFM, Cp(5/6)	VHM, Cp(5/6)	4.693×10-6	1.028×10-5	41	0.456	0.025	1.000
**PHM, Cp(5/6)**	**VHM, Cp(5/6)**	**-3.288×10-5**	**8.134×10-6**	**41**	**-4.042**	**-0.174**	**0.007**
**VFM, Cp(1/2)**	**PHM, Cp(1/2)**	**2.152×10-5**	**4.284×10-6**	**41**	**5.023**	**0.114**	**< 0.001**
VFM, Cp(1/2)	VHM, Cp(1/2)	1.036×10-5	4.601×10-6	41	2.252	0.055	0.387
PHM, Cp(1/2)	VHM, Cp(1/2)	-1.116×10-5	4.062×10-6	41	-2.747	-0.059	0.151

Table S2.4: Post Hoc Comparisons—Age group for main RM Anova 13–30 Hz Range

Post Hoc Comparisons - Age group							
		Mean Difference	SE	df	t	Cohen's d	pHolm
**3-4 years-old**	**5-6 years-old**	**1.001×10-4**	**4.122×10-5**	**41**	**2.429**	**0.531**	**0.039**
**3-4 years-old**	**7-8 years-old**	**1.371×10-4**	**4.512×10-5**	**41**	**3.040**	**0.727**	**0.012**
5-6 years-old	7-8 years-old	3.702×10-5	3.484×10-5	41	1.062	0.196	0.294

S2.5.1: RM Anova for the 3–4 years-old in the 13–30 Hz Range.

Within Subjects Effects						
Cases	Sum of Squares	df	Mean Square	F	p	$\eta^2 p$
**Condition**	**6.177×10-7**	**2**	**3.088×10-7**	**14.77**	**< 0.001**	**0.678**

**Region**	**6.628×10-6**	**5**	**1.326×10-6**	**38.43**	**< 0.001**	**0.846**
**Condition × Region**	**5.465×10-7**	**10**	**5.465×10-8**	**6.114**	**< 0.001**	**0.466**

Table S2.5.1.2: Post Hoc Comparisons—Condition for RM Anova for the 3–4 years-old in the 13–30 Hz Range

Post Hoc Comparisons - Condition							
		Mean Difference	SE	df	t	Cohen's d	pHolm
**VFM**	**PHM**	**1.457×10-4**	**2.987×10-5**	**7**	**4.880**	**0.812**	**0.005**
VFM	VHM	1.480×10-5	2.704×10-5	7	0.547	0.082	0.601
**PHM**	**VHM**	**-1.309×10-4**	**3.148×10-5**	**7**	**-4.159**	**-0.729**	**0.008**

Table S2.5.1.3: Post Hoc Comparisons—Condition × Region for RM Anova for the 3–4 years-old in the 13–30 Hz Range

Post Hoc Comparisons - Condition × Region							
		Mean Difference	SE	df	t	Cohen's d	pHolm
VFM, F(3/4)	PHM, F(3/4)	3.422×10-4	1.033×10-4	7	3.314	1.906	0.437
VFM, F(3/4)	VHM, F(3/4)	7.785×10-5	8.299×10-5	7	0.938	0.434	1.000
PHM, F(3/4)	VHM, F(3/4)	-2.644×10-4	6.621×10-5	7	-3.993	-1.472	0.241
VFM, FC(5/6)	PHM, FC(5/6)	3.136×10-4	7.864×10-5	7	3.988	1.747	0.241
VFM, FC(5/6)	VHM, FC(5/6)	-3.422×10-5	6.473×10-5	7	-0.529	-0.191	1.000
PHM, FC(5/6)	VHM, FC(5/6)	-3.478×10-4	1.148×10-4	7	-3.029	-1.937	0.632
**VFM, FC(1/2)**	**PHM, FC(1/2)**	**4.529×10-5**	**5.941×10-6**	**7**	**7.623**	**0.252**	**0.017**
VFM, FC(1/2)	VHM, FC(1/2)	4.147×10-6	7.436×10-6	7	0.558	0.023	1.000
PHM, FC(1/2)	VHM, FC(1/2)	-4.115×10-5	1.008×10-5	7	-4.081	-0.229	0.225
VFM, C(3/4)	PHM, C(3/4)	8.849×10-5	2.548×10-5	7	3.473	0.493	0.378
VFM, C(3/4)	VHM, C(3/4)	2.252×10-5	2.956×10-5	7	0.762	0.125	1.000
PHM, C(3/4)	VHM, C(3/4)	-6.598×10-5	1.684×10-5	7	-3.918	-0.367	0.248
VFM, CP(5/6)	PHM, CP(5/6)	5.610×10-5	2.564×10-5	7	2.188	0.312	1.000
VFM, CP(5/6)	VHM, CP(5/6)	1.608×10-5	2.625×10-5	7	0.613	0.090	1.000
PHM, CP(5/6)	VHM, CP(5/6)	-4.002×10-5	1.198×10-5	7	-3.340	-0.223	0.435
VFM, CP(1/2)	PHM, CP(1/2)	2.872×10-5	9.512×10-6	7	3.019	0.160	0.632
VFM, CP(1/2)	VHM, CP(1/2)	2.402×10-6	7.146×10-6	7	0.336	0.013	1.000
PHM, CP(1/2)	VHM, CP(1/2)	-2.632×10-5	8.800×10-6	7	-2.991	-0.147	0.632

Table S2.5.2: RM Anova for the 5–6 years-old in the 13–30 Hz Range

Within Subjects Effects						
Cases	Sum of Squares	df	Mean Square	F	p	η^2p

**Condition**	**4.452×10-7**	**2**	**2.226×10-7**	**13.99**	**< 0.001**	**0.389**
**Region**	**7.763×10-6 [a]**	**5[a]**	**1.553×10-6 [a]**	**83.04[a]**	**< 0.001[a]**	**0.791**
**Condition × Region**	**3.164×10-7**	**10**	**3.164×10-8**	**5.945**	**< 0.001**	**0.213**

Table S2.5.2.2: Post Hoc Comparisons—Condition for RM Anova for the 5–6 years-old in the 13–30 Hz Range

Post Hoc Comparisons - Condition							
		Mean Difference	SE	df	t	Cohen's d	pHolm
**VFM**	**PHM**	**6.190×10-5**	**1.101×10-5**	**22**	**5.625**	**0.445**	**< 0.001**
VFM	VHM	-1.338×10-5	1.659×10-5	22	-0.807	-0.096	0.429
**PHM**	**VHM**	**-7.528×10-5**	**1.718×10-5**	**22**	**-4.381**	**-0.542**	**< 0.001**

Table S2.5.2.3: Post Hoc Comparisons—Condition × Region for RM Anova for the 5–6 years-old in the 13–30 Hz Range

Post Hoc Comparisons - Condition × Region							
		Mean Difference	SE	df	t	Cohen's d	pHolm
**VFM, F(3/4)**	**PHM, F(3/4)**	**1.654×10-4**	**3.772×10-5**	**22**	**4.384**	**1.190**	**0.012**
VFM, F(3/4)	VHM, F(3/4)	-2.882×10-5	4.175×10-5	22	-0.690	-0.207	1.000
PHM, F(3/4)	VHM, F(3/4)	-1.942×10-4	5.371×10-5	22	-3.616	-1.397	0.058
VFM, FC(5/6)	PHM, FC(5/6)	9.547×10-5	2.662×10-5	22	3.586	0.687	0.058
VFM, FC(5/6)	VHM, FC(5/6)	-2.237×10-5	4.432×10-5	22	-0.505	-0.161	1.000
**PHM, FC(5/6)**	**VHM, FC(5/6)**	**-1.178×10-4**	**3.138×10-5**	**22**	**-3.755**	**-0.848**	**0.045**
VFM, FC(1/2)	PHM, FC(1/2)	2.821×10-5	1.170×10-5	22	2.410	0.203	0.519
VFM, FC(1/2)	VHM, FC(1/2)	-1.856×10-6	1.153×10-5	22	-0.161	-0.013	1.000
PHM, FC(1/2)	VHM, FC(1/2)	-3.006×10-5	1.056×10-5	22	-2.846	-0.216	0.235
**VFM, C(3/4)**	**PHM, C(3/4)**	**4.155×10-5**	**9.307×10-6**	**22**	**4.465**	**0.299**	**0.010**
VFM, C(3/4)	VHM, C(3/4)	-1.699×10-5	1.449×10-5	22	-1.172	-0.122	1.000
**PHM, C(3/4)**	**VHM, C(3/4)**	**-5.854×10-5**	**1.542×10-5**	**22**	**-3.797**	**-0.421**	**0.042**
VFM, CP(5/6)	PHM, CP(5/6)	2.533×10-5	1.057×10-5	22	2.397	0.182	0.519
VFM, CP(5/6)	VHM, CP(5/6)	-1.430×10-5	1.263×10-5	22	-1.132	-0.103	1.000
**PHM, CP(5/6)**	**VHM, CP(5/6)**	**-3.963×10-5**	**1.064×10-5**	**22**	**-3.725**	**-0.285**	**0.047**
VFM, CP(1/2)	PHM, CP(1/2)	1.549×10-5	4.823×10-6	22	3.211	0.111	0.117
VFM, CP(1/2)	VHM, CP(1/2)	4.050×10-6	5.970×10-6	22	0.678	0.029	1.000
PHM, CP(1/2)	VHM, CP(1/2)	-1.144×10-5	4.868×10-6	22	-2.350	-0.082	0.519

Table S2.5.3: RM Anova for the 7–8 years-old in the 13–30 Hz Range

Within Subjects Effects

Cases	Sum of Squares	df	Mean Square	F	p	η^2p
**Condition**	**2.205×10-7**	**2**	**1.103×10-7**	**6.929**	**0.004**	**0.366**
**Region**	**3.717×10-6** [a]	**5**[a]	**7.435×10-7** [a]	**69.28**[a]	**< 0.001**[a]	**0.852**
Condition × Region	9.720×10-8	10	9.720×10-9	1.474	0.157	0.109

Table S2.5.3.2: Post Hoc Comparisons—Condition for RM Anova for the 7–8 years-old in the 13–30 Hz Range;

Post Hoc Comparisons - Condition							
		Mean Difference	SE	df	t	Cohen's d	pHolm
**VFM**	**PHM**	**7.015×10-5**	**1.901×10-5**	**12**	**3.691**	**0.544**	**0.009**
VFM	VHM	1.164×10-5	2.332×10-5	12	0.499	0.090	0.627
**PHM**	**VHM**	**-5.852×10-5**	**1.785×10-5**	**12**	**-3.278**	**-0.454**	**0.013**

Figure S2: Mean amplitude spectra in the 8–13 Hz range at channels C3 and C4, shown separately for each age subgroup (3–4, 5–6, 7–8 years) and experimental condition (PHM right hand, PHM left hand, VFM, VHM). Spectra are averaged across all participants within each age subgroup at the first longitudinal time point

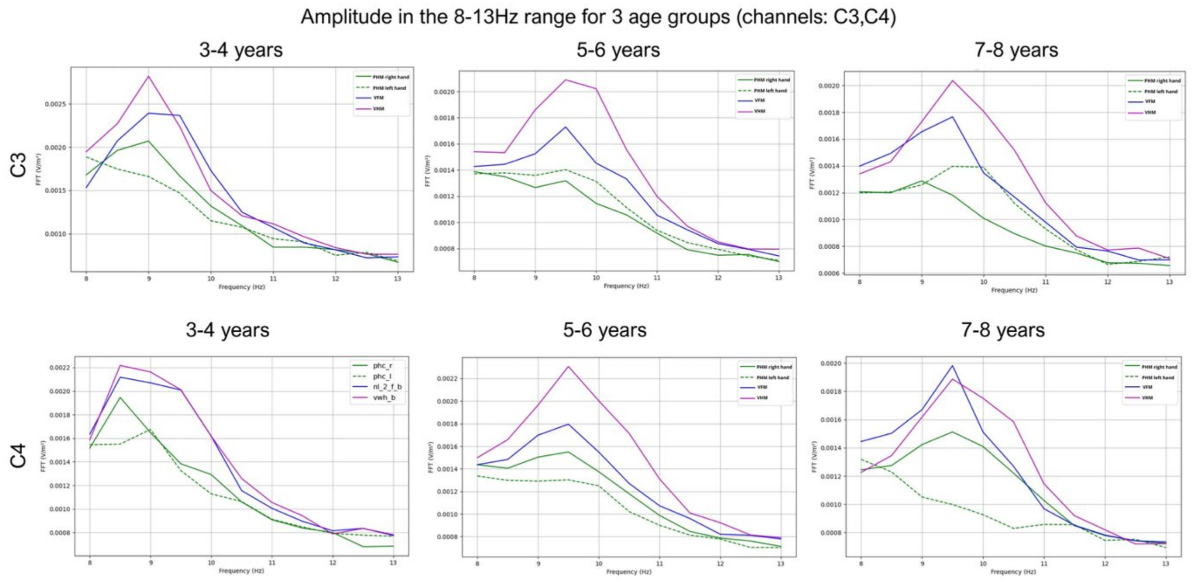

Figure S3: Mean amplitude spectra in the 13–30 Hz range at channels C3 and C4, shown separately for each age subgroup (3–4, 5–6, 7–8 years) and experimental condition (PHM right hand, PHM left hand, VFM, VHM). Spectra are averaged across all participants within

each age subgroup at the first longitudinal time point.

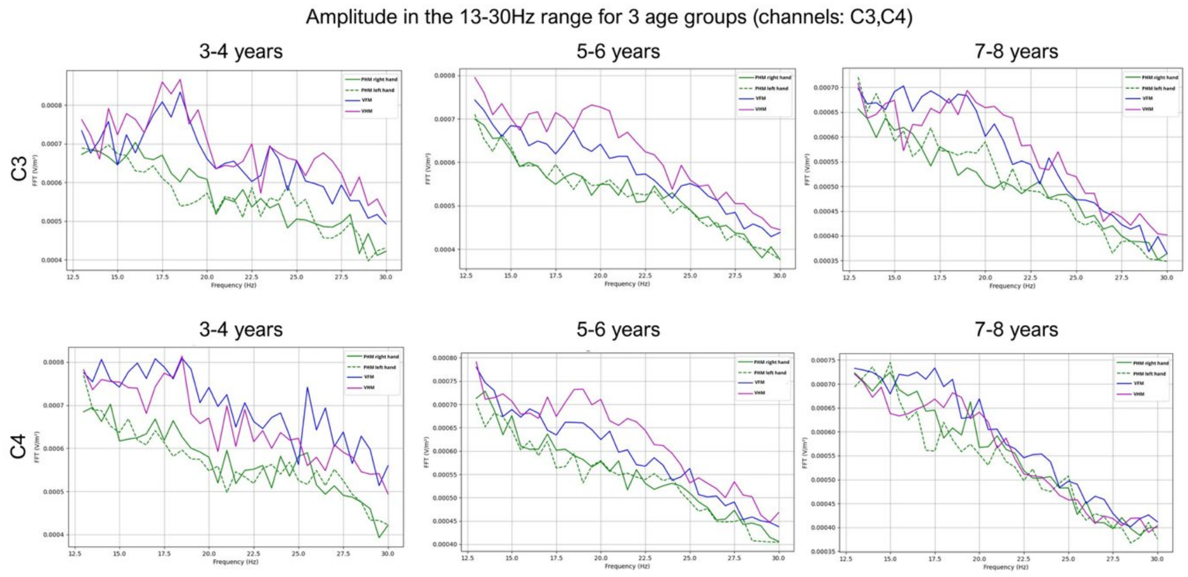

Analysis of Lateralization Effects during Right- and Left-Hand clenching

Table S3.1: RM Anova of Lateralization Effects during Right- and Left-Hand clenching in the 8–13 Hz Range;

Within Subjects Effects						
Cases	Sum of Squares	df	Mean Square	F	p	η^2p
**Lateralization**	**2.017×10-6 [a]**	**2[a]**	**1.008×10-6 [a]**	**15.54[a]**	**< 0.001[a]**	**0.275**
Lateralization × Age group	1.684×10-7 [a]	4[a]	4.209×10-8 [a]	0.649[a]	0.629[a]	0.031
Hemisphere	7.278×10-8	1	7.278×10-8	1.237	0.273	0.029
Hemisphere × Age group	5.677×10-8	2	2.839×10-8	0.482	0.621	0.023
**Region**	**1.350×10-5 [a]**	**5[a]**	**2.700×10-6 [a]**	**38.08[a]**	**< 0.001[a]**	**0.482**
Region × Age group	3.907×10-7 [a]	10[a]	3.907×10-8 [a]	0.551[a]	0.852[a]	0.026
**Lateralization × Hemisphere**	**9.910×10-7**	**2**	**4.955×10-7**	**14.41**	**< 0.001**	**0.260**
Lateralization × Hemisphere × Age group	1.222×10-7	4	3.055×10-8	0.888	0.475	0.042
**Lateralization × Region**	**1.239×10-6 [a]**	**10[a]**	**1.239×10-7 [a]**	**6.764[a]**	**< 0.001[a]**	**0.142**
Lateralization × Region × Age group	4.179×10-7 [a]	20[a]	2.089×10-8 [a]	1.140[a]	0.305[a]	0.053
**Lateralization × Hemisphere × Region**	**6.592×10-7 [a]**	**10[a]**	**6.592×10-8 [a]**	**5.557[a]**	**< 0.001[a]**	**0.119**
Lateralization × Hemisphere × Region ×	1.912×10-7 [a]	20[a]	9.561×10-9 [a]	0.806[a]	0.707[a]	0.038

Age group						
Within Subjects Effects						
Cases	Sum of Squares	df	Mean Square	F	p	η²p
**Age group**	**1.057×10-5**	**2**	**5.287×10-6**	**3.831**	**0.030**	**0.157**

Table S3.1.2: Post Hoc Comparisons—Lateralization for RM Anova Lateralization Effects in the 8–13 Hz Range

Post Hoc Comparisons - Lateralization							
		Mean Difference	SE	df	t	Cohen's d	pHolm
**VFM**	**PHM right**	**8.557×10-5**	**2.024×10-5**	**41**	**4.229**	**0.327**	**< 0.001**
**VFM**	**PHM left**	**7.996×10-5**	**1.992×10-5**	**41**	**4.013**	**0.306**	**< 0.001**
PHM right	PHM left	-5.617×10-6	8.838×10-6	41	-0.636	-0.021	0.529

Table S3.1.3: Post Hoc Comparisons—Lateralization × Hemisphere for RM Anova Lateralization Effects in the 8–13 Hz Range

Post Hoc Comparisons - Lateralization × Hemisphere							
		Mean Difference	SE	df	t	Cohen's d	pHolm
**VFM, Left**	**PHM right, Left**	**1.251×10-4**	**2.610×10-5**	**41**	**4.795**	**0.478**	**< 0.001**
VFM, Left	PHM left, Left	5.280×10-5	2.443×10-5	41	2.162	0.202	0.256
VFM, Left	VFM, Right	-6.575×10-6	1.862×10-5	41	-0.353	-0.025	1.000
VFM, Left	PHM right, Right	3.943×10-5	2.150×10-5	41	1.834	0.151	0.295
**VFM, Left**	**PHM left, Right**	**1.005×10-4**	**2.150×10-5**	**41**	**4.676**	**0.384**	**< 0.001**
**PHM right, Left**	**PHM left, Left**	**-7.234×10-5**	**1.801×10-5**	**41**	**-4.018**	**-0.276**	**0.002**
**PHM right, Left**	**VFM, Right**	**-1.317×10-4**	**2.837×10-5**	**41**	**-4.643**	**-0.503**	**< 0.001**
**PHM right, Left**	**PHM right, Right**	**-8.571×10-5**	**2.099×10-5**	**41**	**-4.083**	**-0.328**	**0.002**
PHM right, Left	PHM left, Right	-2.460×10-5	1.433×10-5	41	-1.716	-0.094	0.295
PHM left, Left	VFM, Right	-5.937×10-5	2.791×10-5	41	-2.127	-0.227	0.256
PHM left, Left	PHM right, Right	-1.337×10-5	2.092×10-5	41	-0.639	-0.051	1.000
PHM left, Left	PHM left, Right	4.774×10-5	1.930×10-5	41	2.473	0.182	0.141
VFM, Right	PHM right, Right	4.600×10-5	2.185×10-5	41	2.106	0.176	0.256
**VFM, Right**	**PHM left, Right**	**1.071×10-4**	**2.165×10-5**	**41**	**4.947**	**0.409**	**< 0.001**
**PHM right, Right**	**PHM left, Right**	**6.111×10-5**	**1.259×10-5**	**41**	**4.853**	**0.234**	**< 0.001**

Table S3.1.4a: Post Hoc Comparisons—Lateralization × Hemisphere × Region for RM Anova Lateralization Effects in the 8–13 Hz Range. Different experimental conditions within the same hemisphere and region

Post Hoc Comparisons - Lateralization × Hemisphere × Region							
		Mean	SE	df	t	Cohen's	p

		Difference				d	Holm
VFM, Left, F(3/4)	PHM right, Left, F(3/4)	8.480×10-5	3.468×10-5	41	2.445	0.324	1.000
VFM, Left, F(3/4)	PHM left, Left, F(3/4)	6.656×10-5	3.606×10-5	41	1.846	0.254	1.000
PHM right, Left, F(3/4)	PHM left, Left, F(3/4)	-1.825×10-5	2.039×10-5	41	-0.895	-0.070	1.000
VFM, Right, F(3/4)	PHM right, Right, F(3/4)	3.950×10-5	4.065×10-5	41	0.972	0.151	1.000
VFM, Right, F(3/4)	PHM left, Right, F(3/4)	5.565×10-5	4.272×10-5	41	1.302	0.213	1.000
PHM right, Right, F(3/4)	PHM left, Right, F(3/4)	1.614×10-5	1.316×10-5	41	1.226	0.062	1.000
VFM, Left, Fc(5/6)	PHM right, Left, Fc(5/6)	3.317×10-5	3.777×10-5	41	0.878	0.127	1.000
VFM, Left, Fc(5/6)	PHM left, Left, Fc(5/6)	8.402×10-6	4.086×10-5	41	0.206	0.032	1.000
PHM right, Left, Fc(5/6)	PHM left, Left, Fc(5/6)	-2.477×10-5	1.210×10-5	41	-2.048	-0.095	1.000
VFM, Right, Fc(5/6)	PHM right, Right, Fc(5/6)	2.935×10-5	5.085×10-5	41	0.577	0.112	1.000
VFM, Right, Fc(5/6)	PHM left, Right, Fc(5/6)	6.092×10-5	4.554×10-5	41	1.338	0.233	1.000
PHM right, Right, Fc(5/6)	PHM left, Right, Fc(5/6)	3.157×10-5	1.590×10-5	41	1.985	0.121	1.000
VFM, Left, Fc(1/2)	PHM right, Left, Fc(1/2)	9.529×10-5	2.399×10-5	41	3.971	0.364	0.109
VFM, Left, Fc(1/2)	PHM left, Left, Fc(1/2)	9.413×10-6	3.049×10-5	41	0.309	0.036	1.000
PHM right, Left, Fc(1/2)	PHM left, Left, Fc(1/2)	-8.588×10-5	2.620×10-5	41	-3.278	-0.328	0.702
VFM, Right, Fc(1/2)	PHM right, Right, Fc(1/2)	4.328×10-5	3.305×10-5	41	1.309	0.165	1.000
**VFM, Right, Fc(1/2)**	**PHM left, Right, Fc(1/2)**	**1.395×10-4**	**3.062×10-5**	**41**	**4.554**	**0.533**	**0.020**
**PHM right, Right, Fc(1/2)**	**PHM left, Right, Fc(1/2)**	**9.617×10-5**	**1.935×10-5**	**41**	**4.971**	**0.368**	**0.006**
**VFM, Left, C(3/4)**	**PHM right, Left, C(3/4)**	**3.376×10-4**	**5.837×10-5**	**41**	**5.783**	**1.290**	**< 0.001**
VFM, Left, C(3/4)	PHM left, Left, C(3/4)	1.375×10-4	5.741×10-5	41	2.395	0.525	1.000
PHM right, Left, C(3/4)	PHM left, Left, C(3/4)	-2.001×10-4	4.794×10-5	41	-4.173	-0.765	0.061
VFM, Right, C(3/4)	PHM right, Right, C(3/4)	1.377×10-4	4.802×10-5	41	2.867	0.526	1.000
**VFM, Right, C(3/4)**	**PHM left, Right, C(3/4)**	**2.754×10-4**	**5.102×10-5**	**41**	**5.398**	**1.053**	**0.002**
PHM right, Right, C(3/4)	PHM left, Right, C(3/4)	1.377×10-4	3.502×10-5	41	3.932	0.526	0.121
VFM, Left, Cp(5/6)	PHM right, Left, Cp(5/6)	7.144×10-5	3.152×10-5	41	2.267	0.273	1.000
VFM, Left, Cp(5/6)	PHM left, Left, Cp(5/6)	2.259×10-5	2.581×10-5	41	0.875	0.086	1.000
PHM right, Left, Cp(5/6)	PHM left, Left, Cp(5/6)	-4.885×10-5	1.679×10-5	41	-2.910	-0.187	1.000
VFM, Right, Cp(5/6)	PHM right, Right, Cp(5/6)	4.267×10-6	2.101×10-5	41	0.203	0.016	1.000
VFM, Right, Cp(5/6)	PHM left, Right, Cp(5/6)	5.009×10-5	2.181×10-5	41	2.297	0.191	1.000
PHM right, Right, Cp(5/6)	PHM left, Right, Cp(5/6)	4.583×10-5	1.539×10-5	41	2.978	0.175	1.000
VFM, Left, Cp(1/2)	PHM right, Left, Cp(1/2)	1.286×10-4	3.204×10-5	41	4.013	0.491	0.096
VFM, Left, Cp(1/2)	PHM left, Left, Cp(1/2)	7.232×10-5	2.737×10-5	41	2.642	0.276	1.000
PHM right, Left, Cp(1/2)	PHM left, Left, Cp(1/2)	-5.625×10-5	1.849×10-5	41	-3.043	-0.215	1.000
VFM, Right, Cp(1/2)	PHM right, Right, Cp(1/2)	2.193×10-5	2.173×10-5	41	1.009	0.084	1.000
VFM, Right, Cp(1/2)	PHM left, Right, Cp(1/2)	6.114×10-5	2.474×10-5	41	2.471	0.234	1.000

PHM right, Right, Cp(1/2)	PHM left, Right, Cp(1/2)	3.922×10-5	1.319×10-5	41	2.973	0.150	1.000

Table S3.1.4b: Post Hoc Comparisons—Lateralization × Hemisphere × Region for RM ANOVA Lateralization Effects in the 8–13 Hz Range. Different hemispheres within the same experimental condition and region

Post Hoc Comparisons - Lateralization × Hemisphere × Region							
		Mean Difference	SE	df	t	Cohen's d	p Holm
VFM, Left, F(3/4)	VFM, Right, F(3/4)	-3.255×10-5	3.534×10-5	41	-0.921	-0.124	1.000
PHM right, Left, F(3/4)	PHM right, Right, F(3/4)	-7.785×10-5	4.208×10-5	41	-1.850	-0.297	1.000
PHM left, Left, F(3/4)	PHM left, Right, F(3/4)	-4.346×10-5	3.976×10-5	41	-1.093	-0.166	1.000
VFM, Left, Fc(5/6)	VFM, Right, Fc(5/6)	-5.147×10-5	4.710×10-5	41	-1.093	-0.197	1.000
PHM right, Left, Fc(5/6)	PHM right, Right, Fc(5/6)	-5.529×10-5	3.753×10-5	41	-1.473	-0.211	1.000
PHM left, Left, Fc(5/6)	PHM left, Right, Fc(5/6)	1.045×10-6	3.538×10-5	41	0.030	0.004	1.000
VFM, Left, Fc(1/2)	VFM, Right, Fc(1/2)	8.398×10-6	2.282×10-5	41	0.368	0.032	1.000
PHM right, Left, Fc(1/2)	PHM right, Right, Fc(1/2)	-4.361×10-5	3.257×10-5	41	-1.339	-0.167	1.000
PHM left, Left, Fc(1/2)	PHM left, Right, Fc(1/2)	1.384×10-4	3.387×10-5	41	4.088	0.529	0.079
VFM, Left, C(3/4)	VFM, Right, C(3/4)	3.638×10-5	4.293×10-5	41	0.847	0.139	1.000
PHM right, Left, C(3/4)	PHM right, Right, C(3/4)	-1.635×10-4	4.038×10-5	41	-4.049	-0.625	0.088
PHM left, Left, C(3/4)	PHM left, Right, C(3/4)	1.743×10-4	5.424×10-5	41	3.213	0.666	0.831
VFM, Left, Cp(5/6)	VFM, Right, Cp(5/6)	-3.710×10-7	3.448×10-5	41	-0.011	-0.001	1.000
PHM right, Left, Cp(5/6)	PHM right, Right, Cp(5/6)	-6.755×10-5	2.352×10-5	41	-2.872	-0.258	1.000
PHM left, Left, Cp(5/6)	PHM left, Right, Cp(5/6)	2.713×10-5	2.324×10-5	41	1.167	0.104	1.000
VFM, Left, Cp(1/2)	VFM, Right, Cp(1/2)	1.639×10-7	2.861×10-5	41	0.006	6.263× 10-4	1.000
PHM right, Left, Cp(1/2)	PHM right, Right, Cp(1/2)	-1.065×10-4	2.681×10-5	41	-3.971	-0.407	0.109
PHM left, Left, Cp(1/2)	PHM left, Right, Cp(1/2)	-1.102×10-5	1.819×10-5	41	-0.606	-0.042	1.000

Table S3.1.5: Post Hoc Comparisons—Age group for RM Anova Lateralization Effects in the 8–13 Hz Range

Post Hoc Comparisons - Age group							
		Mean Difference	SE	df	t	Cohen's d	pHolm
3-4 years-old	5-6 years-old	1.528×10-4	8.036×10-5	41	1.901	0.584	0.129
**3-4 years-old**	**7-8 years-old**	**2.435×10-4**	**8.798×10-5**	**41**	**2.768**	**0.931**	**0.025**
5-6 years-old	7-8 years-old	9.070×10-5	6.794×10-5	41	1.335	0.347	0.189

Table S3.2: RM Anova of Lateralization Effects during Right- and Left-Hand clenching in the 13–30 Hz Range

Within Subjects Effects						
Cases	Sum of Squares	df	Mean Square	F	p	η²p
**Lateralization**	**1.808×10-6** [a]	**2**[a]	**9.039×10-7**[a]	**38.65**[a]	**< 0.001**[a]	**0.485**
Lateralization × Age group	1.780×10-7 [a]	4[a]	4.451×10-8[a]	1.903[a]	0.118[a]	0.085
Hemisphere	2.883×10-8	1	2.883×10-8	1.162	0.287	0.028
Hemisphere × Age group	1.108×10-7	2	5.540×10-8	2.233	0.120	0.098
**Region**	**1.992×10-5** [a]	**5**[a]	**3.984×10-6**[a]	**144.0**[a]	**< 0.001**[a]	**0.778**
**Region × Age group**	**1.404×10-6** [a]	**10**[a]	**1.404×10-7**[a]	**5.073**[a]	**< 0.001**[a]	**0.198**
Lateralization × Hemisphere	1.887×10-8 [a]	2[a]	9.434×10-9[a]	0.796[a]	0.455[a]	0.019
Lateralization × Hemisphere × Age group	1.321×10-8 [a]	4[a]	3.303×10-9[a]	0.279[a]	0.891[a]	0.013
**Lateralization × Region**	**1.407×10-6** [a]	**10**[a]	**1.407×10-7**[a]	**10.45**[a]	**< 0.001**[a]	**0.203**
Lateralization × Region × Age group	4.122×10-7 [a]	20[a]	2.061×10-8[a]	1.531[a]	0.067[a]	0.069
Lateralization × Hemisphere × Region	2.599×10-8 [a]	10[a]	2.599×10-9[a]	0.330[a]	0.973[a]	0.008
Lateralization × Hemisphere × Region × Age group	7.635×10-8 [a]	20[a]	3.818×10-9[a]	0.485[a]	0.972[a]	0.023
Within Subjects Effects						
Cases	Sum of Squares	df	Mean Square	F	p	η²p
**Age group**	**2.735×10-6**	**2**	**1.368×10-6**	**4.239**	**0.021**	**0.171**

Table S3.2.2: Post Hoc Comparisons—Lateralization for RM Anova Lateralization Effects in the 13–30 Hz Range

Post Hoc Comparisons - Lateralization							
		Mean Difference	SE	df	t	Cohen's d	pHolm
**VFM**	**PHM right**	**7.368×10-5**	**1.164×10-5**	**41**	**6.327**	**0.473**	**< 0.001**
**VFM**	**PHM left**	**8.256×10-5**	**1.215×10-5**	**41**	**6.797**	**0.530**	**< 0.001**
PHM right	PHM left	8.885×10-6	5.978×10-6	41	1.486	0.057	0.145

Table S3.2.3: Post Hoc Comparisons—Age group for RM Anova Lateralization Effects in the 13–30 Hz Range

Post Hoc Comparisons - Age group

		Mean Difference	SE	df	t	Cohen's d	pHolm
3-4 years-old	5-6 years-old	8.664×10-5	3.886×10-5	41	2.230	0.556	0.063
**3-4 years-old**	**7-8 years-old**	**1.229×10-4**	**4.254×10-5**	**41**	**2.888**	**0.788**	**0.018**
5-6 years-old	7-8 years-old	3.622×10-5	3.285×10-5	41	1.103	0.232	0.277

Relationship between Mu Rhythm Modulation Index and Speech

Table S4.1: Correlation between PLS-5 scores and Modulation Index PHM in the 8–13 Hz range

PLS-5 speech measures	PHM Modulation Index Channel	Spearman`s rho	p(FDR)
Auditory Comprehension	F3	-0,10151458	0,692474956
Auditory Comprehension	FC5	0,100780297	0,692474956
Auditory Comprehension	FC1	-0,317944398	0,375561882
Auditory Comprehension	C3	-0,198072751	0,58694588
Auditory Comprehension	CP5	-0,1070217	0,692474956
Auditory Comprehension	CP1	-0,050481934	0,829896272
Auditory Comprehension	CP6	-0,078751817	0,751880433
Auditory Comprehension	CP2	-0,212941976	0,544378661
Auditory Comprehension	C4	-0,097292454	0,692474956
Auditory Comprehension	FC6	-0,244883272	0,48946327
Auditory Comprehension	FC2	-0,037081275	0,864327021
Auditory Comprehension	F4	-0,227444058	0,505442594
Expressive Communication	F3	-0,357897008	0,375561882
Expressive Communication	FC5	-0,066256831	0,783960652
Expressive Communication	FC1	-0,448013639	0,182308989
Expressive Communication	C3	-0,190144256	0,594478821
Expressive Communication	CP5	-0,362485431	0,375561882
Expressive Communication	CP1	-0,116362412	0,692474956
Expressive Communication	CP6	-0,157291147	0,606976065
Expressive Communication	CP2	-0,182619242	0,600858108
Expressive Communication	C4	-0,278976129	0,439384127
Expressive Communication	FC6	-0,152519186	0,606976065
Expressive Communication	FC2	-0,156556999	0,606976065
Expressive Communication	F4	-0,343030517	0,375561882
Total Language	F3	-0,302852529	0,375561882
Total Language	FC5	0,011189585	0,951533406
Total Language	FC1	-0,46629384	0,182308989
Total Language	C3	-0,250573322	0,48946327

Total Language	CP5	-0,301201607	0,375561882
Total Language	CP1	-0,100889698	0,692474956
Total Language	CP6	-0,163074439	0,606976065
Total Language	CP2	-0,228194153	0,505442594
Total Language	C4	-0,249656143	0,48946327
Total Language	FC6	-0,154086084	0,606976065
Total Language	FC2	-0,142162756	0,630228563
Total Language	F4	-0,308722475	0,375561882

Table S4.2: Correlation between PLS-5 scores and Modulation Index PHM in the 13–30 Hz range

PLS-5 speech measures	PHM Modulation Index Channel	Spearman`s rho	p(FDR)
Auditory Comprehension	F3	-0,341625014	0,078026796
Auditory Comprehension	FC5	-0,283249542	0,137017868
**Auditory Comprehension**	**FC1**	**-0,466085928**	**0,021522999**
**Auditory Comprehension**	**C3**	**-0,482423717**	**0,020677854**
Auditory Comprehension	CP5	-0,176778554	0,333106539
Auditory Comprehension	CP1	-0,337953601	0,078026796
Auditory Comprehension	CP6	-0,276457427	0,137017868
**Auditory Comprehension**	**CP2**	**-0,487380125**	**0,020677854**
Auditory Comprehension	C4	-0,348233558	0,078026796
**Auditory Comprehension**	**FC6**	**-0,417990412**	**0,041484894**
Auditory Comprehension	FC2	-0,393759084	0,051525901
**Auditory Comprehension**	**F4**	**-0,619734577**	**0,001862251**
Expressive Communication	F3	-0,441773384	0,136342986
Expressive Communication	FC5	-0,120400224	0,621252994
Expressive Communication	FC1	-0,325227435	0,164250117
Expressive Communication	C3	-0,137285621	0,621252994
Expressive Communication	CP5	-0,104432512	0,621252994
Expressive Communication	CP1	0,009176846	0,960243784
Expressive Communication	CP6	0,124438037	0,621252994
Expressive Communication	CP2	-0,114343506	0,621252994
Expressive Communication	C4	-0,345600034	0,164250117
Expressive Communication	FC6	-0,312012776	0,164250117
Expressive Communication	FC2	-0,323575603	0,164250117
Expressive Communication	F4	-0,351656752	0,164250117
**Total Language**	**F3**	**-0,479134346**	**0,033174474**

Total Language	FC5	-0,191873862	0,390379165
Total Language	FC1	-0,423002987	0,063439585
Total Language	C3	-0,304320016	0,154924528
Total Language	CP5	-0,140695269	0,482674609
Total Language	CP1	-0,141062141	0,482674609
Total Language	CP6	-0,034302497	0,852147857
Total Language	CP2	-0,253324859	0,242742717
Total Language	C4	-0,392369206	0,076931258
Total Language	FC6	-0,374025625	0,076931258
Total Language	FC2	-0,367605372	0,076931258
**Total Language**	**F4**	**-0,513069972**	**0,032093166**
